# Supplementary material for: The biopsychosocial factors associated with development of chronic musculoskeletal pain. An umbrella review and meta-analysis of observational systematic reviews
Source: PLoS One. 2024 Apr 1;19(4):e0294830. doi: 10.1371/journal.pone.0294830 (PMC10984407; doi:10.1371/journal.pone.0294830)
Supplement: S2 File — (DOCX) [file pone.0294830.s002.docx]

**S3 File.** Methods for Application of GRADE for an Epidemiological Umbrella Review

This guidance is based on the Cochrane Handbook for Systematic Reviews of Interventions [1] and GRADE guidance for the assessment of evidence of prognostic factors [2]. As per the GRADE guidelines by Foroutan [2], all studies in this umbrella review started with a rating of ‘high’ as all eligible reviews included are of prospective longitudinal cohort studies. The confidence of findings were then downgraded by one or two levels as considered for risk of bias, inconsistency, imprecision, indirectness, and publication bias. We also considered three domains for rating up certainty: large effect, plausible residual confounding bias, and dose response.

**Risk of bias**

Risk of bias was assessed using the AMSTAR 2 critical appraisal tool whereby an overall confidence rating is given to what extent the systematic review provides an accurate and comprehensive summary of the results of the available studies that address the question of interest [3]. All reviews where confidence in findings were found to be either ‘critically low’ or ‘low’ were considered to be high risk of bias. Those found to be ‘moderate’ or ‘high’ confidence in findings were considered as ‘moderate’ or ‘low’ risk of bias respectively and were further scrutinised to assess whether multivariable or sub-group analysis of findings had been conducted to further assess the rigour of the finding [2]. For example, one review found that female sex increased odds of developing CMP, however a further subgroup analysis of female participants recruited only from studies that did not financially compensate participants demonstrated that female sex was not associated with CMP. Further, a sub-group analysis of female participants recruited only from studies which financially compensated participants demonstrated a marked increase in odds of developing CMP. Multivariable analysis with all other findings did not change the association of female sex with CMP. This highlights the importance of multivariable and sub-group analysis. Confidence in findings may have been downgraded if this was not considered. In all cases, an overall judgement was made as to whether downgrading is warranted on the basis of review limitations likely having influenced reported results. Where most reviews for each finding have one critical flaw downgrading of one level was considered, further critical flaws or serious limitations warranted consideration for downgrading two levels. Confidence in findings may not have been downgraded in instances where review limitations were considered unlikely to have influenced the overall reported result [1, 2].

**Inconsistency**

Inconsistency was expected and therefore *a priori* hypotheses were developed which may explain significant heterogeneity between findings e.g. absence of multivariable or sub-group analysis or absence of quantitative data/meta-analysis [2]. Inconsistency can be assessed as non-contextualised whereby downgrading certainty of confidence in findings is unnecessary so long as all reviews suggest some degree of association (e.g., all odds ratios >1.0) whatever the magnitude. Alternatively, when assessed in the contextualised setting inconsistency may be considered if the magnitude of association may influence clinical decision making. For the purpose of this umbrella review inconsistency was assessed within the non-contextualised setting because of the likelihood that a combination of multiple biopsychosocial factors increases risk of development of CMP rather than any single overarching factor (as discussed in the main manuscript), and therefore magnitude of effect of any single factor was small. Therefore, so long as all reviews and/or quantitative findings of primary studies suggested some degree of association confidence in findings was not downgraded [2]. This was determined through visual inspection of the funnel plot for each meta-analysis and consideration of overall findings of reviews. Confidence in findings may have been downgraded if there were conflicting findings between reviews or primary quantitative data used for meta-analysis, and substantial unexplained heterogeneity remained after consideration of *a priori* hypotheses. However, this may not have been downgraded if observed inconsistency is unlikely to have influenced the overall reported result; for example, if the contribution to the pooled estimate was small [1, 2].

*In the absence of meta-analysis*

Inconsistency was judged by evaluating the consistency of the direction of association of the factor with CMP and any indication of the magnitude of association as reported within reviews. The same considerations were made regarding explainable or unexplainable heterogeneity as outlined above.

**Imprecision**

Similar to inconsistency, imprecision can be considered in the non-contextualised or contextualised settings depending on the threshold of the magnitude of effect required to influence clinical decision making [2]. For the same reason outlined previously, imprecision has been assessed within a non-contextualised framework. Imprecision was determined if the lower boundary of the 95% confidence intervals of the meta-analysis was <1.0 for ratio effect sizes [2]. In these instances, findings were considered to be imprecise and may have been downgraded. Additionally, confidence in findings may have been downgraded if the quantitative data from original studies were not reported, if overall sample sizes were not reported or if the sample was <400 [4]. In all cases, an overall judgement was made; confidence in findings may not have been downgraded where any observed imprecision was unlikely to have influenced the overall reported result; for example if the contribution of this sample to the pooled estimate was small [1, 2].

*In the absence of meta-analysis*

The above considerations were instead applied to quantitative data from the original studies if presented in the review. If no quantitative findings were presented, then the findings were considered imprecise and may have been downgraded.

**Indirectness**

Indirectness was determined through the clarity and thoroughness of the PICOS elements of eligibility criteria [1, 2]. Additionally, consideration was given to how well populations and outcomes studied in reviews correlate to the population and outcome of interest outlined in this umbrella review. For example, one review which fulfilled our inclusion criteria stated “studies investigating psychological processes in chronic (>3 months) back pain” as part of their exclusion criteria; however, included 5 original studies where there is no data on time since onset thus raising the likelihood of inclusion of participants with chronic back pain. Confidence in findings may have been downgraded in these instances. In all cases an overall judgement was made on the likelihood of observed indirectness having influenced the overall reported result. Confidence in findings may not have been downgraded where observed indirectness is thought unlikely to have influenced the overall reported result [1, 2].

**Publication bias**

Cochrane and GRADE guidance suggest authors of systematic reviews assess for the presence of publication bias through the use of statistical tests (e.g. Begg’s, Debray’s, Peter’s or Egger’s tests) or/and visual exploration of the funnel plot [1, 2]. However, positive tests for publication bias may not necessarily require downgrading of confidence in findings. For example, a positive Eggers test may be disregarded if the funnel plot demonstrates that the missing studies would not significantly affect the level of association [2]. Assessment of publication bias was planned with Egger’s regression test and visual inspection of a funnel plot, however this was not appropriate due to the number of reviews or original studies included in each meta-analysis falling below the recommended 10 studies/reviews for this method [1]. Therefore, publication bias was assessed through alternative means as outlined below.

*For all findings including those with and without meta-analysis*

Publication bias (small study bias) was assessed according to Cochrane guidance for the assessment of publication bias within umbrella reviews in the absence of statistical methods [5, 6]. Publication bias was considered present when review authors did not assess for, or appropriately interpret, the impact of publication bias on the reported result; if original studies included in reviews consist of only positive studies with a small sample size; if most studies have been commercially funded or if conflicts of interest are apparent; or if the search strategy was unavailable or did not meet the criteria for an effective search strategy (according to Peer Review of Electronic Search Strategies guidance [7]) [1, 4-6, 8]. In these instances, an overall judgement was made and confidence in findings may not have been downgraded where this was considered unlikely to have affected the overall reported result [1, 2].

**Rating up certainty**

Consideration of dose response, large effect or for the nature of plausible confounding biases were considered for rating up confidence in findings [2]. For the purpose of this umbrella review, rating up certainty of evidence required agreement of at least three of the five co-authors (MD, AR, JM, AS, NH) with reasons for doing so recorded, however no instances of rating up certainty occurred.

**Double downrating across domains**

There are instances of crossover between domains of GRADE as our risk of bias assessment with AMSTAR 2 includes considerations of information which is relevant for both indirectness (e.g., PICOS) and publication bias (e.g. search strategy, funding). Therefore, we scrutinised and rated indirectness and publication bias in more detail under their respective domains as described, however if downrating had already occurred due to the same identified methodological flaws within our risk of bias assessment, certainty was not downrated again.

1. Higgins J, Thomas J, Chandler J, Cumpston M, Li T, Page M, et al. Cochrane Handbook for Systematic reviews of Interventions version 6.3 (updated February 2022). Cochrane 2022. 2022;Available from <www.training.cochrane.org/handbook>.

2. Foroutan F, Guyatt G, Zuk V, Vandvik PO, Alba AC, Mustafa R, et al. GRADE Guidelines 28: Use of GRADE for the assessment of evidence about prognostic factors: rating certainty in identification of groups of patients with different absolute risks. Journal of Clinical Epidemiology. 2020;121:62-70. doi: 10.1016/j.jclinepi.2019.12.023.

3. Shea BJ, Reeves BC, Wells G, Thuku M, Hamel C, Moran J, et al. AMSTAR 2: a critical appraisal tool for systematic reviews that include randomised or non-randomised studies of healthcare interventions, or both. BMJ. 2017;358:j4008. doi: 10.1136/bmj.j4008.

4. Guyatt GH, Oxman AD, Kunz R, Brozek J, Alonso-Coello P, Rind D, et al. GRADE guidelines 6. Rating the quality of evidence--imprecision. J Clin Epidemiol. 2011;64(12):1283-93. Epub 2011/08/16. doi: 10.1016/j.jclinepi.2011.01.012. PubMed PMID: 21839614.

5. Pollock M, Fernandes R, Becker L, Pieper D, Hartling L. Chapter V: Overviews of Reviews. In: Higgins JPT, Thomas J, Chandler J, Cumpston M, Li T, Page MJ, Welch VA (editors). *Cochrane Handbook for Systematic Reviews of Interventions* version 6.3 (updated February 2022). Cochrane, 2022. Available from <www.training.cochrane.org/handbook>. (accessed 24/03/2022). 2022.

6. Meader N, King K, Llewellyn A, Norman G, Brown J, Rodgers M, et al. A checklist designed to aid consistency and reproducibility of GRADE assessments: development and pilot validation. Syst Rev. 2014;3:82. Epub 2014/07/25. doi: 10.1186/2046-4053-3-82. PubMed PMID: 25056145; PubMed Central PMCID: PMCPMC4124503.

7. McGowan J, Sampson M, Salzwedel DM, Cogo E, Foerster V, Lefebvre C. PRESS Peer Review of Electronic Search Strategies: 2015 Guideline&#xa0;Statement. Journal of Clinical Epidemiology. 2016;75:40-6. doi: 10.1016/j.jclinepi.2016.01.021.

8. Murad MH, Chu H, Lin L, Wang Z. The effect of publication bias magnitude and direction on the certainty in evidence. BMJ Evid Based Med. 2018;23(3):84-6. Epub 2018/04/14. doi: 10.1136/bmjebm-2018-110891. PubMed PMID: 29650725; PubMed Central PMCID: PMCPMC5969367.
