# Supplementary material for: The biopsychosocial factors associated with development of chronic musculoskeletal pain. An umbrella review and meta-analysis of observational systematic reviews
Source: PLoS One. 2024 Apr 1;19(4):e0294830. doi: 10.1371/journal.pone.0294830 (PMC10984407; doi:10.1371/journal.pone.0294830)
Supplement: S1 Table — (DOCX) [file pone.0294830.s003.docx]

**S2 Table.** Ovid MEDLINE Search Strategy

| ***Line*** | ***Associated factors*** |
| --- | --- |
| 1 | Validat*.mp. or Predict*.ti. or Rule*.mp. or (Predict* and (Outcome* or Risk* or Model*)).mp. or ((History or Variable* or Criteria or Scor* or Characteristic* or Finding* or Factor*) and (Predict* or Model* or Decision* or Identif* or Prognos*)).mp. or (Decision*.mp. and ((Model* or Clinical*).mp. or Logistic Models/)) or (Prognostic and (History or Variable* or Criteria or Scor* or Characteristic* or Finding* or Factor* or Model*)).mp. |
| 2 | (Stratification or discrimination or discriminate or c statistic or auc or calibration or indices or algorithm or multivariable).tw. |
| 3 | (Area under adj1 curve).tw. |
| 4 | Predict*.tw. or predictive value of tests/ or Scor*.tw. or Observ*.tw. or observer variation/ |
| 5 | Roc curve/ or Forecasting/ or prognosis/ or exp probability/ or exp epidemiologic factors/ or exp regression analysis/ |
| 6 | (Predict* or forecasting or Prognos* or probability or determin* or moderator or effect modifi* or regression analys?s* or logistic regression or logistic model* or odds ratio or risk ratio or relative risk or risk factor* or protective factor* or Cox model* or hazard* model* or hazard ratio or reason* or theme* or drive* or indicator* or sign* or link* or correlat* or cause* or factor* or affordance or constraint or obstacle or impediment or enabler or inhibit* or stimulat* or facilitate* or barrier*).tw,kf. |
| 7 | (dependenc* or mobility aid or crutch* or walking stick* or walking frame* or zimmer frame* or rollator frame* or wheelchair* or obese or obesity or overweight or high BMI or underweight or low BMI or age or older or elderly or comorbidit* or mental health or emotion* or depression or anxiety or distress or headache* or poorly controlled pain or pain intensity or education or pre existing pain or catastophi?* or female or gender or male or psychological or biopsychosocial or psychosocial or smok*OR sensory disturbance or sensory deficit or altered sensation or paraesthesia or fear* or avoid* or occupation or return* work or genetics or genes or swelling or reduced range motion or reduced range movement or reduced ROM or loss range motion or loss range movement or loss ROM or restricted range motion or restricted range movement or restricted ROM or limited weight bearing or reduced weight bearing or ability weight bear or re-injury or stress or passive coping strategies or reduced self efficacy or low self efficacy or somati?ation or resilience or concern* or physical activity or motivation or persistan* or guarding or guarded or socioeconomic status or alcohol or high* income or low* income or social support or support* network or propriocept* or joint position sense).tw,kf. |
| 8 | (Concomitant adj3 pain).tw,kf. |
| 9 | (Poor* adj2 health).tw,kf. |
| 10 | or/1-9 |
| ***Line*** | ***Chronic pain*** |
| 11 | Chronic pain/ OR ((Persistent or chronic or recurrent or continu* or lasting or long or intractable or endur*) adj2 pain).tw,kf. |
| 12 | (exp Outcome Assessment, Health Care/ or patient outcome assessment/ or treatment outcome/ or program evaluation/ or disability evaluation/ or follow-up studies/) and Chronic pain/ |
| 13 | ((Poor* or reduced or worse or inferior) adj2 outcome*).tw,kf. |
| 14 | or/11-13 |
| ***Line*** | ***All musculoskeletal conditions*** |
| 15 | Knee.tw,kf. or Knee/ or exp knee joint/ or anterior cruciate ligament/ |
| 16 | exp knee injuries/ or Knee dislocation/ or Osteoarthritis, knee/ or Medial Collateral Ligament, Knee/in or Posterior cruciate ligament/in or menisci, tibial/in or Patellofemoral pain syndrome/ or patella/in or patella ligament/in or quadriceps muscle/in |
| 17 | "sprains and strains"/ and knee.tw,kf. |
| 18 | ((knee or patellofemoral or tibiofemoral or patella* or prepatella* or tibia* or fibula* or meniscus or menisci or collateral ligament or medial patellofemoral ligament or cruciate ligament or hamstring* or quadricep* or semitendinosus or semimembranosus or biceps femoris or popliteus or popliteal or iliotibial band or chondromalacia or plica) adj3 (sprain* or strain* or myalgia or tear* or bursitis or tend?nopathy or tend?nitis or tend?nosis or tenosynovitis or rupture or injur* or impair* or disorder or dysfunction or trauma or arthritis or osteoarthritis or syndrome)).tw,kf. |
| 19 | (PFP or PFPS or chondromalacia patella).tw,kf. |
| 20 | (Ankle or foot or toe*).tw,kf. or Ankle/ or exp foot joints/ or lateral ligament, ankle/ |
| 21 | Ankle injuries/ or lateral ligament, ankle/in or achilles tendon/in or exp foot joints/in or fasciitis, plantar/ or heel spur/ or exp metatarsalgia/ or posterior tibial tendon dysfunction/ or medial tibial stress syndrome/ |
| 22 | "sprains and strains"/ and (ankle or foot).tw,kf. |
| 23 | ((ankle or foot or talus or talar or calcaneu* or cuboid or navicular or cuneiform or metatarsal or tarsal or hallux or malleolus or malleolar or talofibular ligament or calcaneofibular ligament or deltoid ligament or tibiotalar ligament or tibiocalcaneal ligament or tibionavicular ligament or achilles or calf or calves or gastroc* or soleus or tibialis or peroneus or peroneal* or digitorum or hallucis or digiti minimi or lumbrical* or interosseus or interossei or retinaculum) adj3 (sprain* or strain* or myalgia or tear* or bursitis or tend?nopathy or tend?nitis or tend?nosis or tenosynovitis or rupture or impingement or injur* or impair* or disorder or dysfunction or trauma or arthritis or osteoarthritis or syndrome)).tw,kf. |
| 24 | (plantar fasciitis or plantar fascia pain).tw,kf. |
| 25 | Shoulder.tw,kf. or Shoulder/ or shoulder joint/ or rotator cuff/ |
| 26 | Shoulder pain/ or shoulder impingement syndrome/ or shoulder joint/in or shoulder injuries/ or rotator cuff injuries/ or shoulder dislocation/ |
| 27 | "sprains and strains"/ and shoulder.tw,kf. |
| 28 | ((Shoulder or glenohumeral or GHJ or humerus or humeral or clavicle or clavicular or scapula* or acromioclavicular or ACJ or scapulothoracic or subacrom* or rotator cuff or supraspinatus or infraspinatus or subscapularis or teres minor or teres major or trapezius or deltoid* or bicep* or bicipital or latissimus dorsi or rhomboid* or labrum or labral or glenoid or supraglenoid or infraglenoid) adj3 (sprain* or strain* or myalgia or tear* or bursitis or tend?nopathy or tend?nitis or tend?nosis or tenosynovitis or impingement or rupture or injur* or impair* or disorder or dysfunction or trauma or arthritis or osteoarthritis or syndrome)).tw,kf. |
| 29 | (Elbow or forearm or wrist or hand or thumb or finger).tw,kf. or Elbow joint/ or exp hand joints/ or exp upper extremity/ |
| 30 | exp hand joints/in or upper extremity/in or exp elbow tendinopathy/ or exp hand injuries/ or De Quervain disease/ |
| 31 | ((Elbow* or forearm* or arm* or wrist* or hand* or finger* or thumb* or radioulnar or metacarpal or interphalangeal or phalanx or thenar or hypothenar or humerus or olecranon or radius or radial or ulna or scaphoid or lunate or triquetrum or pisiform or trapezium or trapezoid or capitate or hamate or scapholunate ligament or collateral ligament or Bicep* or Bicipital or coracobrachialis or brachioradialis or brachialis or tricep* or carpi radialis or carpi ulnaris or pronator teres or digitorum or Pronator quadratus or palmaris longus or supinator or pollicis or lumbrical* or digiti minimi or interosseus or interossei or triangular cartilage or triangular fibrocartilage complex or TFCC) adj3 (sprain* or strain* or myalgia or tear* or bursitis or tend?nopathy or tend?nitis or tend?nosis or tenosynovitis or rupture or impingement or injur* or impair* or disorder or dysfunction or trauma or arthritis or osteoarthritis or syndrome)).tw,kf. |
| 32 | (de quervain* or golfers elbow or tennis elbow).tw,kf. |
| 33 | ((medial or lateral) adj1 (epicondylitis or epicondylalgia or epicondylopathy or epicondylosis or tendinopathy or elbow pain)).tw,kf. |
| 34 | (Thoracic or mid back or middle back or upper back).tw,kf. or Thoracic vertebrae/ or thorax/ or Intercostal muscles/ or intermediate back muscles/ or paraspinal muscles/ |
| 35 | (osteoarthritis/ or osteoarthritis, spine/ or myofascial pain syndromes/ or exp spinal osteophytosis/ or spondylolisthesis/ or intervertebral disc displacement/) and (thoracic or mid back or middle back or upper back).tw,kf. |
| 36 | Tietze's Syndrome/ |
| 37 | ((thoracic or thoracic spin* or T spin* or thoracic wall or thorax or thoracolumbar or thoracic intervertebral disc or thoracic disc or thoracic muscle* or erector spin* or intercostal or costovertebral or costotransverse or costochondral or rib or pectoral or T1 or T2 or T3 or T4 or T5 or T6 or T7 or T8 or T9 or T10 or T11 or T12) adj3 (strain* or impingement or compression or stenosis or narrowing or injur* or impair* or disorder or dysfunction or trauma or arthritis or osteoarthritis or syndrome or spondylitis or spondylolysis or spondylolisthesis or spondylosis or bulge or herniation or prolapse or slipped)).tw,kf. |
| 38 | (Lumb?r or low* back).tw,kf. or Lumbar vertebrae/ or exp back/ or exp back muscles/ or sacroiliac joint/ |
| 39 | (Osteoarthritis/ or osteoarthritis, spine/ or myofascial pain syndromes/ or exp osteophytosis/ or spondylolisthesis/ or intervertebral disc displacement/) and (lumb?r or back).tw,kf. |
| 40 | back pain/ or low back pain/ or exp back injuries/ |
| 41 | ((low* back or lumb?r or sacroiliac or lumbar facet or SIJ or L1 or L2 or L3 or L4 or L5 or S1) adj3 (strain* or impingement or compression or stenosis or narrowing or injur* or impair* or disorder or dysfunction or trauma or arthritis or osteoarthritis or syndrome or spondylitis or spondylolysis or spondylolisthesis or spondylosis or bulge or herniation or prolapse or slipped)).tw,kf. |
| 42 | non specific low* back pain.tw,kf. |
| 43 | (Hip or pelvi?).tw,kf. or Hip/ or hip joint/ or exp pelvis/ |
| 44 | Osteoarthritis, hip/ or Femoroacetabular impingement/ or hip dislocation/ or hip injuries/ or pelvic girdle pain/ or piriformis syndrome/ |
| 45 | "sprains and strains"/ and hip.tw,kf. |
| 46 | ((hip or labrum or labral or greater trochanter* or GT or lesser trochanter* or acetabulum or femur or femoral or groin or thigh or hip flexor* or pelvi? or iliac or ilium or ischium or ischial or pubi? or ASIS or anterior superior iiliac spine or rectus femoris or vastus medialis or vastus lateralis or vastus intermedius or iliacus or psoas major or iliopsoas or gluteus or gluteal or adductor or piriformis) adj3 (sprain* or strain* or myalgia or tear* or bursitis or tend?nopathy or tend?nitis or tend?nosis or tenosynovitis or rupture or impingement or injur* or impair* or disorder or dysfunction or trauma or arthritis or osteoarthritis or syndrome)).tw,kf. |
| 47 | (FAI or PGP).tw,kf. |
| 48 | (neck or cervical or temporomandibular).tw,kf. or Neck/ or neck muscles/ or cervical plexus/ or exp cervical vertebrae/ or atlanto-axial joint/ or atlanto-occipital joint/ or temporomandibular joint/ |
| 49 | (osteoarthritis/ or osteoarthritis, spine/ or myofascial pain syndromes/ or exp spinal osteophytosis/ or spondylolisthesis/ or intervertebral disc displacement/) and (cervical or neck).tw,kf. |
| 50 | exp neck pain/ or temporomandibular joint dysfunction syndrome/ or exp neck injuries/ or cervical rib syndrome/ or torticollis/ |
| 51 | ((neck or odontoid or cervical or occip* or atlant* or cervical disc or cervical interverteb* or cervical facet or C1 or C2 or C3 or C4 or C5 or C6 or C7 or C8) adj3 (strain* or impingement or compression or stenosis or narrowing or injur* or impair* or disorder or dysfunction or trauma or arthritis or osteoarthritis or syndrome or spondylitis or spondylolysis or spondylolisthesis or spondylosis or bulge or herniation or prolapse or slipped)).tw,kf. |
| 52 | Musculoskeletal pain/ OR myofascial pain syndromes/ |
| 53 | or/15-52 |
| ***Line*** | ***Systematic review/meta-analysis*** |
| 54 | (systematic review or meta analysis).tw,kf. |
| ***Line*** | ***All strings combined*** |
| 55 | 10 and 14 and 53 and 54 |
