# Supplementary material for: The biopsychosocial factors associated with development of chronic musculoskeletal pain. An umbrella review and meta-analysis of observational systematic reviews
Source: PLoS One. 2024 Apr 1;19(4):e0294830. doi: 10.1371/journal.pone.0294830 (PMC10984407; doi:10.1371/journal.pone.0294830)
Supplement: S2 Table — (DOCX) [file pone.0294830.s004.docx]

**S5 Table.** Review characteristics

| **Author** | **No. primary studies** | **Sample** | **Age** | **Exposures** | **Duration MSK pain at baseline** | **Length of follow up** | **Population details** |
| --- | --- | --- | --- | --- | --- | --- | --- |
| **Agnello et al. (2010)** | 10 | 2484 | 18-65 | Any potentially predictive factors | <6 weeks | >6 months | Low back pain. Traumatic sustained in occupational setting. 6 cohorts recruited from primary care setting; 1 cohort recruited from compensation claimants. Includes studies conducted in Netherlands, USA, and Norway. |
| **Buscemi et al. (2019)** | 3 | 22,575 | 18+ | Baseline levels of stress or recall of previous stressful events | No MSK pain, or <6 weeks | 1-6 years | Spinal pain. No details on setting. Includes studies conducted in Canada, Sweden and USA. |
| **Chou and Shekelle (2010)** | 20 | 10,842 | 18+ | Any potentially predictive factors | <8 weeks | 3-24 months | Low back pain. One third of studies included patients in workers' compensation settings, two thirds of studies included patients in clinical care settings. Countries of studies not stated. |
| **Dai et al. (2021)** | 4 | 38,188 | 20+ | Smoking | No MSK pain | 7-15 years | Low back pain. Members of the general public who do or do not smoke without MSK pain at baseline. Studies conducted in Australia, Finland, Norway and The Netherlands. |
| **Fayad et al. (2004)** | 26 | 39,046 | 18+ | Any potentially predictive factors | “Pre-chronic” | Not stated | Low back pain. 19 studies recruited patients (n=8761), 5 studies recruited employees (n=28,941), 2 studies recruited nurses (n=1344). Includes studies conducted in Denmark, Sweden, UK, Canada, USA, France, Netherlands, New Zealand, and Germany. |
| **Goldsmith et al. (2012)** | 6 | 443 | 18-70 | Cold hyperalgesia | <1 month | 6-36 months | Whiplash associated disorder as a result of road traffic collision. Includes studies conducted in Denmark and Australia. Study settings not stated. |
| **Iles et al. (2009)** | 7 | 2514 | 18+ | Poor recovery expectations | 0-58 days | 3-24 months | Low back pain. 1 study recruited in primary care (N= 354), 3 studies recruited workers filing compensation claims (N= 570), 1 study recruited from occupational health clinic (N= 522), 1 study recruited workers reporting sick leave in primary care (N= 596), 1 study recruited workers claiming insurance (N= 1068). Includes studies conducted in USA, Netherlands, and Canada. |
| **Jadhakhan et al. (2023)** | 7 | 1695 | 18-70 | Post-traumatic stress symptoms | <3 months | 3-12 months | Whiplash associated disorder as a result of any traumatic event. Three studies conducted in Australia, three conducted in Denmark, one conducted in Sweden. Four studies recruited from emergency departments, one from both emergency departments and general practice, one from emergency department and primary care advertisement and one using emergency department register. |
| **Lang et al. (2012)** | 50 | 362,605 | 18+ | Psychosocial work stressors in industrialised countries | No MSK pain | 6 months – 28 years | General MSK conditions experienced in occupational settings. Idiopathic or traumatic onset. Includes studies conducted in Denmark, Netherlands, Sweden, USA, Belgium, France, Switzerland, Australia, Norway, UK, Finland, and Canada. 34 studies recruited employees from various working backgrounds, 13 studies recruited members of the general population, 3 studies recruited ‘users of computers’. |
| **Pincus et al. (2002)** | 25 | 4184 | 18+ | At least one psychological variable | <3 months | Until chronic | Low back pain. Includes studies conducted in UK, Sweden, USA, Norway, Australia, Canada, and Finland. 9 studies recruited from primary care, 10 studies recruited from "clinics", 6 studies recruited from workplace. |
| **Struyf et al. (2016)** | 4 | 754 | 18+ | Any potentially predictive factors | “Acute” or “sub-acute” | 6-36 months | Shoulder pain. Idiopathic onset. 1 study recruited workers; 3 studies recruited patients. Not stated countries of studies. |
| **Walton et al. (2009)** | 14 | 3193 | 18+ | Any potentially predictive factors | <3 weeks | 6-24 months | Whiplash associated disorder acquired from road traffic collisions. 6 studies recruited from ED, 3 studies recruited from both primary care and ED, 1 study recruited from insurance claims, 1 study recruited from tertiary care. Included studies conducted in UK, Sweden, Norway, Canada, Netherlands, Denmark, and Switzerland. |
| **Walton et al. (2013)** | 9 | 1121 | 18+ | Any potentially predictive factors | <3 weeks | 6-12 months | Whiplash associated disorder acquired from road traffic collisions. 1 study recruited from ED, 1 recruited from ED and GPs, 1 recruited from ED, primary care and general public, 1 recruited from ED and hospital clinic. Included studies conducted in Denmark, Sweden, Australia, and Norway. |
