# Supplementary material for: The biopsychosocial factors associated with development of chronic musculoskeletal pain. An umbrella review and meta-analysis of observational systematic reviews
Source: PLoS One. 2024 Apr 1;19(4):e0294830. doi: 10.1371/journal.pone.0294830 (PMC10984407; doi:10.1371/journal.pone.0294830)
Supplement: S3 Table — (DOCX) [file pone.0294830.s005.docx]

**S6 Table.** Definitions of identified biopsychosocial domains and factors

| **Physical health** | These factors are underpinned by determinants of physical health of an individual with MSK pain |
| --- | --- |
| *Smoking* | ‘Smoking’ is described as “ever smoked” [1] or “current smoker” [1, 2]. Descriptions regarding how much one has smoked (e.g., pack years) were not provided |
| *Poorer general health* | ‘Poorer general health’ was determined based on self-perception of health identified through patient reported outcome measures such as the General Health Questionnaire-28 [3] or a four-tiered scale on self-perception of health as poor, fair, good or excellent. Based on this, poorer general health’ is defined as self-perception of poorer general health identified through an appropriate patient reported outcome measure or through self-perception of health as ‘poor’ or ‘fair’ |
| *High BMI* | ‘High BMI’ was defined as a BMI higher than 25 or 27. Based on this, for the purpose of this umbrella review ‘high BMI’ is defined as higher than 25 |
| *History of the same MSK pain* | ‘History of the same MSK pain’ is defined as having previous experience of musculoskeletal pain in the same region of the body e.g., a new onset of whiplash associated disorder and a history of neck pain |
| **Psychological factors** | These factors are underpinned by their psychological impact upon an individual with MSK pain |
| *Fear avoidance* | ‘Fear avoidance’ was determined by included reviews through patient reported outcome measures such as the Fear Avoidance Beliefs Questionnaire [4] with scores reported as low, moderate or high. This questionnaire was based on the fear avoidance model first described by Lethem [5] as ‘the avoidance of movements or activities based on the fear of pain or (re)injury’. Based on this, ‘fear avoidance’ is defined as high levels of avoidance of movement or activity based on fear as determined through an appropriate patient reported outcome measure |
| *Post-trauma stress symptoms* | ‘Post trauma stress symptoms’ was defined according to the International Classification of Diseases clusters of symptoms [6] and the Diagnostic and Statistical Manual of Mental Disorders [7] for post-trauma stress symptoms. The review only included studies which adhered to these definitions. Based on this, ‘post-trauma stress symptoms’ is defined as the presence of the following symptoms: re-experiencing, avoidance, hyperarousal, negative alterations in mood, or distorted sense of current threat, as a result of musculoskeletal trauma, identified through an appropriate outcome measure |
| *Stressful childhood experiences* | ‘Stressful childhood experiences’ was determined through the use of outcome measures such as the List of Threatening Experiences [8] and includes events such as ‘>2 weeks hospitalisation’ and ‘parental unemployment’. Based on this, ‘stressful childhood experiences’ is defined as any stressful event which occurred during childhood identified through an appropriate outcome measure |
| *Poorer psychological health* | ‘Poorer psychological health’ was described by included reviews as depression, anxiety or psychological distress determined through patient reported outcome measures including questionnaires (e.g., Hospital Anxiety and Depression Score) and numerical rating scales (0-10). Included reviews did not differentiate between specific disorders and used broader descriptions such as ‘poor psychological status’ instead. Based on these descriptions, ‘poorer psychological health’ is defined as the presence of anxiety, depression, or psychological distress identified through an appropriate outcome measure |
| *Poorer recovery expectations* | ‘Poorer recovery expectations’ was determined through patient reported outcome measures orientated around the individual’s expectation of recovery of function within a set time frame. Based on this, ‘poorer recovery expectations’ is defined as poorer expectations of return to pre-morbid function within four weeks of onset of musculoskeletal pain |
| *Somatisation* | ‘Somatisation’ was determined by included reviews through the sub-scales of the Symptom Checklist-90 [9] reported as either higher or lower levels of somatisation. The authors of this questionnaire describe somatisation as ‘distress arising from perceptions of bodily dysfunction’ determined through levels of ‘botheredness’ by symptoms such as feeling faint or weak. Based on this, ‘somatisation’ is defined as higher levels of distress arising from perception of bodily dysfunction in response to musculoskeletal pain determined through an appropriate patient reported outcome measure |
| *Stress* | ‘Stress’ was determined through the Perceived Stress Scale [10] which, through a series of questions, identifies levels of stress as low, moderate or high over the prior one-month period. Based on this, ‘stress’ is defined as high levels of stress for the one-month period prior to the onset of musculoskeletal pain |
| *Depression* | ‘Depression’ was not clearly defined by included reviews and it is not clear how depression was measured in the original studies. It is therefore not appropriate to offer a clear definition for the purpose of this umbrella review |
| *Catastrophising* | ‘Catastrophising’ was determined by included reviews through the sub-scales of the Coping Strategies Questionnaire [11]. The authors of this questionnaire describe catastrophising as ‘an exaggerated negative mental state during an actual or anticipated pain experience’ determined through level of agreement with statements such as “I feel I can’t stand it anymore” [11]. Based on this, ‘catastrophising’ is defined as an exaggerated negative mental state in response to musculoskeletal pain determined through an appropriate patient reported outcome measure |
| *Poorer coping strategies* | ‘Poorer coping strategies’ was not clearly defined by included reviews and it is not clear how poorer coping strategies was measured in original studies. It is therefore not appropriate to offer a clear definition for the purpose of this umbrella review |
| **Psychosocial factors** | These factors are underpinned by a social factor which presents a psychological impact upon individuals with MSK pain |
| *Lower job satisfaction* | ‘Lower job satisfaction’ was determined through patient reported outcome measures such as a numerical rating scale (0-10) or a level of agreement scale (e.g., very low, low, moderately or completely) about job satisfaction. Based on this, ‘lower job satisfaction’ is defined as lower satisfaction from a job measured as <5/10 on a numerical rating scale (10 being the most satisfied) or “very low” or “low” on a four-tiered level of agreement scale |
| *Poorer support networks* | ‘Poorer support networks’ was described by included reviews as low social support, low co-worker or supervisor support, or lack of adaptations to job role. Further details regarding definitions or outcome measures used were not provided. From these descriptions, it is also not clear whether poorer support networks existed prior to musculoskeletal pain or in response to the onset of musculoskeletal pain. Based on this, ‘poorer support networks’ is defined as self-perception of low social support or low support at work prior to and/or since onset of musculoskeletal pain |
| *Lower socioeconomic status* | ‘Lower socioeconomic status was described as no higher (university/college) education, lower income, and economic stress. Lower income and economic stress were not clearly defined and it is not clear how these were measured in the original studies. Based on this, for the purpose of this umbrella review ‘lower socioeconomic status’ is defined as no university/college education, or self-perceived low income/financial stress |
| *Financial compensation* | ‘Financial compensation’ was determined as either applying to receive or receiving compensation. Based on this, ‘financial compensation’ is defined as either applying to receive or receiving compensation in relation to musculoskeletal pain |
| *Higher job demands* | ‘Higher job demands’ was determined by one review [1] through a numerical rating scale (0-10) in relation to physical demand, or self-identifying work as requiring regular heavy lifting. However, other reviews [12-14] did not describe higher job demands or provide details of outcome measures used. Based on this, for the purpose of this umbrella review ‘higher job demands’ is defined as self-perception of higher physical workload identified by a score of ≥5/10 on a numerical rating scale (10 being the most physically demanding) or self-identifying as work requiring “regular heavy lifting” |
| *Lower job control* | ‘Lower job control’ was not clearly defined and it is not clear how this was measured in the original studies. It is therefore not appropriate to offer a clear definition for the purpose of this umbrella review |
| *Lower job security* | ‘Lower job security’ was not clearly defined and it is not clear how this was measured in the original studies. It is therefore not appropriate to offer a clear definition for the purpose of this umbrella review |
| *Higher domestic responsibilities* | ‘Higher domestic responsibilities’ was described by the included review as high number of children, single parenthood, or high domestic workload. No further details on these descriptions or outcome measures used were provided. Based on this, for the purpose of this umbrella review ‘higher domestic responsibilities’ is defined as single parenthood, self-perception of high number of children, or self-perception of high domestic workload |
| *Dissatisfaction during leisure activities* | ‘Dissatisfaction during leisure activities’ was not clearly defined and it is not clear how this was measured in the original studies. It is therefore not appropriate to offer a clear definition for the purpose of this umbrella review |
| *Being divorced or widowed without children* | ‘Being divorced or widowed without children’ is not further described by the included review, nor is it clear whether participants were recently divorced or widowed or if this was long-standing. It is therefore not appropriate to offer a clear definition for the purpose of this umbrella review |
| **Symptoms or experiences at or near onset** | These factors are underpinned by the symptoms or experiences of individuals as recorded at baseline data collection. The timeframe ‘at or near onset’ ranges from 0 days – 3 months since onset of MSK pain |
| *High levels of pain at or near onset* | ‘High levels of pain at or near onset’ was determined through numerical rating scales (0-10) or three-tiered verbal descriptors (e.g., low, moderate or severe). Baseline pain intensity was recorded within 8 weeks of onset of musculoskeletal pain. Based on these descriptions, ‘high levels of pain at or near onset’ is defined as a numerical rating of ≥5/10 (10 being the most pain), or verbally described as ‘severe’ (or any equivalent term e.g., extreme) within 8 weeks since onset of musculoskeletal pain |
| *Concomitant pain* | ‘Concomitant pain’ was described as additional pain at baseline. Based on this, for the purpose of this umbrella review ‘concomitant pain’ is defined as experiencing any kind of pain in any other region of the body additional to the [main] musculoskeletal pain |
| *Higher levels of functional impairment at onset* | ‘Higher levels of functional impairment at onset’ was determined by included reviews through functional outcome measures such as the Roland Disability Questionnaire or described using three-tiered verbal descriptors of interference with function from musculoskeletal pain (e.g., low, moderate or high). Based on this, ‘higher levels of functional impairment at onset’ is defined as a high score of functional disability determined through the use of an appropriate outcome measure, or verbally described as having a ‘high’ impact on function at or near onset of musculoskeletal pain |
| *Disturbed sleep since onset* | ‘Disturbed sleep since onset’ is described by the one review which reports on this factor as difficulty sleeping. Further details regarding this definition or outcome measures used were not provided. Based on this, ‘disturbed sleep since onset’ is defined as self-perception of difficulty sleeping since the onset of musculoskeletal pain |
| *Time off work* | ‘Time off work’ was described by included reviews using ranges to describe length of time off of work with the lowest amount of time being described as sick leave <1 week. Based on this, ‘time off work’ is defined as taking any time off work due to musculoskeletal pain |
| *Making physical compensations* | ‘Making physical compensations’ was described by included reviews as absence from sports activities, altering the work-station, or reducing physical load in response to musculoskeletal pain. Based on this, ‘making physical compensations’ is defined as avoiding or modifying usual functional activities or behaviours in response to musculoskeletal pain. An important distinction between this factor and ‘fear avoidance’ is that fear is not reported as part of ‘making physical compensations’ |
| *Cold hyperalgesia* | ‘Cold hyperalgesia’ was not described by the included review and therefore it is not appropriate to offer a clear definition |
| *Sudden onset* | ‘Sudden onset’ was described as the sudden onset of musculoskeletal pain. It is not clear how this was determined. Based on this, ‘sudden onset’ is defined as a sudden onset of musculoskeletal pain |
| *Lack of energy* | ‘Lack of energy’ was not clearly defined by the one review which reports on this factor and it is not clear how lack of energy was measured in the original study. It is therefore not appropriate to offer a clear definition of ‘lack of energy’ |
| **Demographics** | These factors are underpinned by demographic information |
| *Female sex/gender* | ‘Female sex/gender’ was not clearly defined by included reviews and therefore it is not appropriate to offer a clear definition of ‘female sex/gender’. The term ‘Female gender’ was used by two reviews [1, 12] and ‘female sex’ was used by two reviews [15, 16] |
| *Higher age* | Higher age was defined as age higher than 46, 50, or 50-55. Based on this, ‘higher age’ is defined as 46 or above |

1. Chou R, Shekelle P. Will this patient develop persistent disabling low back pain? Jama. 2010;303(13):1295-302. Epub 2010/04/08. doi: 10.1001/jama.2010.344. PubMed PMID: 20371789.

2. Dai Y, Huang J, Hu Q, Huang L, Wu J, Hu J. Association of Cigarette Smoking with Risk of Chronic Musculoskeletal Pain: A Meta-Analysis. Pain Physician. 2021;24(8):495-506. Epub 2021/11/19. PubMed PMID: 34793634.

3. Goldberg DP, Hillier VF. A scaled version of the General Health Questionnaire. Psychological Medicine. 1979;9(1):139-45. Epub 2009/07/09. doi: 10.1017/S0033291700021644.

4. Waddell G, Newton M, Henderson I, Somerville D, Main CJ. A Fear-Avoidance Beliefs Questionnaire (FABQ) and the role of fear-avoidance beliefs in chronic low back pain and disability. Pain. 1993;52(2):157-68. Epub 1993/02/01. doi: 10.1016/0304-3959(93)90127-b. PubMed PMID: 8455963.

5. Lethem J, Slade PD, Troup JDG, Bentley G. Outline of a fear-avoidance model of exaggerated pain perception—I. Behaviour Research and Therapy. 1983;21(4):401-8. doi: <https://doi.org/10.1016/0005-7967(83)90009-8>.

6. Brewin CR, Cloitre M, Hyland P, Shevlin M, Maercker A, Bryant RA, et al. A review of current evidence regarding the ICD-11 proposals for diagnosing PTSD and complex PTSD. Clinical Psychology Review. 2017;58:1-15. doi: <https://doi.org/10.1016/j.cpr.2017.09.001>.

7. Association AP. Diagnostic and statistical manual of mental disorders (5th ed.) American Psychiatric Association. Available from <https://www.psychiatry.org/psychiatrists/practice/dsm>. Accessed 2nd May 2023. 2013.

8. Brugha T, Bebbington P, Tennant C, Hurry J. The List of Threatening Experiences: a subset of 12 life event categories with considerable long-term contextual threat. Psychological Medicine. 1985;15(1):189-94. Epub 2009/07/09. doi: 10.1017/S003329170002105X.

9. Derogatis LR, Lipman RS, Covi L. SCL-90: an outpatient psychiatric rating scale--preliminary report. Psychopharmacol Bull. 1973;9(1):13-28. Epub 1973/01/01. PubMed PMID: 4682398.

10. Cohen S, Kamarck T, Mermelstein R. A global measure of perceived stress. Journal of health and social behavior. 1983:385-96.

11. Rosenstiel AK, Keefe FJ. The use of coping strategies in chronic low back pain patients: relationship to patient characteristics and current adjustment. Pain. 1983;17(1):33-44. Epub 1983/09/01. doi: 10.1016/0304-3959(83)90125-2. PubMed PMID: 6226916.

12. Fayad F, Lefevre-Colau MM, Poiraudeau S, Fermanian J, Rannou F, Wlodyka Demaille S, et al. [Chronicity, recurrence, and return to work in low back pain: common prognostic factors]. Annales de Readaptation et de Medecine Physique. 2004;47(4):179-89. PubMed PMID: 15130717.

13. Lang J, Ochsmann E, Kraus T, Lang JW. Psychosocial work stressors as antecedents of musculoskeletal problems: a systematic review and meta-analysis of stability-adjusted longitudinal studies. Soc Sci Med. 2012;75(7):1163-74. Epub 2012/06/12. doi: 10.1016/j.socscimed.2012.04.015. PubMed PMID: 22682663.

14. Struyf F, Geraets J, Noten S, Meeus M, Nijs J. A multivariable prediction model for the chronification of non-traumatic shoulder pain: A systematic review. Pain Physician. 2016;19(2):1-10. PubMed PMID: 608013657.

15. Agnello A, Brown T, Desroches S, Welling U, Walton D. Can we identify people at risk of non-recovery after acute occupational low back pain? Results of a review and higher-order analysis. Physiotherapy Canada. 2010;62(1):9-16. doi: 10.3138/physio.62.1.9. PubMed PMID: 105126216. Language: English. Entry Date: 20100409. Revision Date: 20150820. Publication Type: Journal Article.

16. Walton DM, Macdermid JC, Giorgianni AA, Mascarenhas JC, West SC, Zammit CA. Risk factors for persistent problems following acute whiplash injury: update of a systematic review and meta-analysis. Journal of Orthopaedic & Sports Physical Therapy. 2013;43(2):31-43. PubMed PMID: 23322093.
