# Supplementary material for: The biopsychosocial factors associated with development of chronic musculoskeletal pain. An umbrella review and meta-analysis of observational systematic reviews
Source: PLoS One. 2024 Apr 1;19(4):e0294830. doi: 10.1371/journal.pone.0294830 (PMC10984407; doi:10.1371/journal.pone.0294830)
Supplement: S4 Table — (DOCX) [file pone.0294830.s006.docx]

**S7 Table.** Descriptive Synthesis of Biopsychosocial Factors

No descriptive synthesis is provided for the following factors as they were only supported by one review: post trauma stress symptoms, stressful childhood experiences, poorer recovery expectations, lower job security, higher domestic responsibilities, dissatisfaction during leisure activities, being divorced or widowed without children, disturbed sleep since onset, cold hyperalgesia, sudden onset, and lack of energy.

| *Smoking* | Both reviews [1, 2] and both of our meta-analyses of odds and likelihood ratios were consistent in demonstrating that smoking is associated with development of CMP. One review is low risk of bias and one review is high risk of bias, however the contribution of the high risk of bias review to the overall sample is small. |
| --- | --- |
| *Poorer general health* | Both reviews [1, 3] and our meta-analysis of likelihood ratios are consistent in demonstrating that poorer general health is associated with development of CMP. |
| *High BMI* | One review [1] and our meta-analysis of likelihood ratios demonstrates that high BMI is not associated with CMP. Whilst the meta-analysis of odds ratios within the other supporting review [4] demonstrates that high BMI is associated, only one of the three primary studies supports this association with an OR of 3.0 which inflates the meta-analysis effect size with wide confidence intervals. Overall, the findings summarised support that high BMI is not associated with development of CMP. |
| *History of the same MSK pain* | Four reviews [1, 4-6], our meta-analysis of meta-analyses of odds ratios, and our meta-analysis of likelihood ratios are consistent in demonstrating that history of the same MSK pain is associated with development of CMP. |
| *Fear avoidance* | Three reviews [1, 3, 6] and our meta-analysis of likelihood ratios are consistent in demonstrating that fear avoidance is associated with development of CMP. |
| *Poorer psychological health* | Three reviews [1, 3, 7] and our meta-analysis of likelihood ratios are consistent in demonstrating that poorer psychological health is associated with development of CMP. |
| *Somatisation* | Three reviews [1, 6, 7] and our meta-analysis of likelihood ratios are consistent demonstrating that somatisation is associated with development of CMP. |
| *Stress* | Both reviews [3, 8] are consistent in demonstrating that stress is associated with development of CMP. Meta-analysis was not possible for this factor, but an odds ratio of one primary study of 1.014 (95% CI 1.01-1.21) is presented within one review [8]. |
| *Depression* | Both reviews [3, 6] are consistent in demonstrating that depression is associated with development of CMP. Meta-analysis was not possible for this factor, but an odds ratio of one primary study of 3.0 (95% CI not provided) is presented within one review [6]. |
| *Catastrophising* | Three reviews [4, 6, 7] and the meta-analysis of odds ratios from one review [4] are consistent in demonstrating that catastrophising is associated with development of CMP. |
| *Poorer coping strategies* | Both reviews [3, 6] are consistent in demonstrating that poorer coping strategies is associated with development of CMP. Meta-analysis was not possible for this factor, but an odds ratio of one primary study of 2.2 (95% CI not provided) is presented within one review [6]. |
| *Lower job satisfaction* | Three reviews [1, 3, 9], our meta-analysis of meta-analyses of odds ratios, and our meta-analysis of likelihood ratios, are consistent in demonstrating that lower job satisfaction is associated with development of CMP. |
| *Poorer support networks* | Three reviews [3, 6, 9] and our meta-analysis of meta-analyses of odds ratios are consistent in demonstrating that poorer support networks is associated with development of CMP. |
| *Lower socioeconomic status* | Four reviews [1, 3, 8, 10], our meta-analysis of meta-analyses of odds ratios, and our meta-analysis of likelihood ratios, are consistent in demonstrating that lower socioeconomic status is associated with development of CMP. |
| *Financial compensation* | Both reviews [1, 3] and our meta-analysis of likelihood ratios are consistent in demonstrating that financial compensation is associated with development of CMP. |
| *Higher job demands* | Four reviews [1, 3, 6, 9], our meta-analysis of meta-analyses of odds ratios, and our meta-analysis of likelihood ratios, are consistent in demonstrating that higher job demands is associated with development of CMP. |
| *Lower job control* | Both reviews [6, 9] and our meta-analysis of meta-analyses of odds ratios are consistent in demonstrating that lower job control is associated with development of CMP. |
| *High levels of pain at or near onset* | Three reviews [1, 3, 10] including meta-analysis of odds ratios within one review [10], and our meta-analysis of likelihood ratios, are consistent in demonstrating that high levels of pain at or near onset is associated with development of CMP. |
| *Concomitant pain* | Both reviews [3, 10] including meta-analysis of odds ratios presented within one review [10] are consistent in demonstrating that concomitant pain is associated with development of CMP. |
| *Higher levels of functional impairment* | Both reviews [1, 3] and our meta-analysis of likelihood ratios are consistent in demonstrating that higher levels of functional impairment is associated with development of CMP. |
| *Time off work* | Both reviews [3, 6] are consistent in demonstrating that time off work is associated with development of CMP. Meta-analysis was not possible for this factor, but odds ratios of two primary studies of 3.3 and 1.8 (95% CI not provided) are presented within one review [6]. |
| *Making physical compensations* | One review demonstrates that making physical compensations is associated with development of CMP [3], whilst one review [6] presents conflicting findings; that reducing shoulder use to <10% of normal is not associated (OR 1.0), but also that continuing to use the shoulder >10% of normal is protective against CMP (OR 0.5). Overall, the findings summarised suggest that making physical compensations is associated with development of CMP. |
| *Female sex/gender* | Four reviews [1, 3, 5, 10], our meta-analysis of meta-analyses of odds ratios, and our meta-analysis of likelihood ratios are consistent in demonstrating that female sex/gender is associated with development of CMP. |
| *Higher age* | An odds ratio meta-analysis of one review [10] demonstrates no association, while our meta-analysis of likelihood ratios, and the three remaining reviews [1, 3, 6] demonstrate association of higher age and development of CMP. Overall, the findings summarised suggest that higher age is associated with development of CMP. |

1. Chou R, Shekelle P. Will this patient develop persistent disabling low back pain? Jama. 2010;303(13):1295-302. Epub 2010/04/08. doi: 10.1001/jama.2010.344. PubMed PMID: 20371789.

2. Dai Y, Huang J, Hu Q, Huang L, Wu J, Hu J. Association of Cigarette Smoking with Risk of Chronic Musculoskeletal Pain: A Meta-Analysis. Pain Physician. 2021;24(8):495-506. Epub 2021/11/19. PubMed PMID: 34793634.

3. Fayad F, Lefevre-Colau MM, Poiraudeau S, Fermanian J, Rannou F, Wlodyka Demaille S, et al. [Chronicity, recurrence, and return to work in low back pain: common prognostic factors]. Annales de Readaptation et de Medecine Physique. 2004;47(4):179-89. PubMed PMID: 15130717.

4. Walton DM, Pretty J, MacDermid JC, Teasel RW. Risk Factors for Persistent Problems Following Whiplash Injury: Results of a Systematic Review and Meta-analysis. Journal of Orthopaedic & Sports Physical Therapy. 2009;39(5):334-50. doi: 10.2519/jospt.2009.2765. PubMed PMID: 19411766.

5. Agnello A, Brown T, Desroches S, Welling U, Walton D. Can we identify people at risk of non-recovery after acute occupational low back pain? Results of a review and higher-order analysis. Physiotherapy Canada. 2010;62(1):9-16. doi: 10.3138/physio.62.1.9. PubMed PMID: 105126216. Language: English. Entry Date: 20100409. Revision Date: 20150820. Publication Type: Journal Article.

6. Struyf F, Geraets J, Noten S, Meeus M, Nijs J. A multivariable prediction model for the chronification of non-traumatic shoulder pain: A systematic review. Pain Physician. 2016;19(2):1-10. PubMed PMID: 608013657.

7. Pincus T, Burton AK, Vogel S, Field AP. A Systematic Review of Psychological Factors as Predictors of Chronicity/Disability in Prospective Cohorts of Low Back Pain. Spine. 2002;27(5):E109-E20. PubMed PMID: 00007632-200203010-00017.

8. Buscemi V, Chang W-J, Liston MB, McAuley JH, Schabrun SM. The role of perceived stress and life stressors in the development of chronic musculoskeletal pain disorders: A systematic review: The Journal of Pain. 2019, pp. No Pagination Specified.; 2019.

9. Lang J, Ochsmann E, Kraus T, Lang JW. Psychosocial work stressors as antecedents of musculoskeletal problems: a systematic review and meta-analysis of stability-adjusted longitudinal studies. Soc Sci Med. 2012;75(7):1163-74. Epub 2012/06/12. doi: 10.1016/j.socscimed.2012.04.015. PubMed PMID: 22682663.

10. Walton DM, Macdermid JC, Giorgianni AA, Mascarenhas JC, West SC, Zammit CA. Risk factors for persistent problems following acute whiplash injury: update of a systematic review and meta-analysis. Journal of Orthopaedic & Sports Physical Therapy. 2013;43(2):31-43. PubMed PMID: 23322093.
