# Supplementary material for: The biopsychosocial factors associated with development of chronic musculoskeletal pain. An umbrella review and meta-analysis of observational systematic reviews
Source: PLoS One. 2024 Apr 1;19(4):e0294830. doi: 10.1371/journal.pone.0294830 (PMC10984407; doi:10.1371/journal.pone.0294830)
Supplement: S5 Table — (DOCX) [file pone.0294830.s007.docx]

**S7 Table.** Primary Data Used for all Meta-analyses

Includes effect size (ES), lower 95% confidence interval (L95), upper 95% confidence interval (U95), and their conversions to the natural log scale (ES ln; L95 ln; U95 ln) and standard error on the natural log scale (SE ln).

| **Fear avoidance LR+** | **ES** | **L95** | **U95** | **ES ln** | **L95 ln** | **U95 ln** | **SE ln** |
| --- | --- | --- | --- | --- | --- | --- | --- |
| Dionne 2007 (3 months) [1] | 4.90 | 3.80 | 6.30 | 1.58 | 1.30 | 1.84 | 0.14 |
| Turner 2006 [2] | 1.70 | 1.50 | 1.90 | 0.53 | 0.41 | 0.64 | 0.06 |
| Swinkels-Meewisse 2006 [3] | 1.80 | 1.10 | 3.00 | 0.59 | 0.10 | 1.10 | 0.26 |
| Truchon 2005 [4] | 1.50 | 0.78 | 2.70 | 0.41 | -0.25 | 0.99 | 0.32 |
| Grotle 2005 [5] | 1.20 | 0.61 | 2.50 | 0.18 | -0.49 | 0.92 | 0.36 |
| Dionne 2007 (1 year) [1] | 2.80 | 2.20 | 2.60 | 1.03 | 0.79 | 0.96 | 0.04 |
| Turner 2008 [6] | 2.00 | 1.60 | 2.40 | 0.69 | 0.47 | 0.88 | 0.10 |
| Grotle 2006 [7] | 1.40 | 0.60 | 3.00 | 0.34 | -0.51 | 1.10 | 0.41 |
| *Meta analysis of studies within Chou* [8] | *2.11* | *1.59* | *2.80* | *0.75* | *0.47* | *1.03* | *0.14* |
| **Fear avoidance LR-** | **ES** | **L95** | **U95** | **ES ln** | **L95 ln** | **U95 ln** | **SE ln** |
| Dionne 2007 (3 months) [1] | 0.3 | 0.22 | 0.41 | -1.20 | -1.54 | -0.89 | 0.17 |
| Turner 2006 [2] | 0.14 | 0.05 | 0.36 | -1.97 | -3.00 | -1.02 | 0.50 |
| Swinkels-Meewisse 2006 [3] | 0.55 | 0.35 | 0.85 | -0.60 | -1.05 | -0.16 | 0.23 |
| Truchon 2005 [4] | 0.73 | 0.59 | 0.9 | -0.31 | -0.53 | -0.11 | 0.11 |
| Grotle 2005 [5] | 0.92 | 0.67 | 1.2 | -0.08 | -0.40 | 0.18 | 0.15 |
| Dionne 2007 (1 year) [1] | 0.38 | 0.28 | 0.52 | -0.97 | -1.27 | -0.65 | 0.16 |
| Turner 2008 [6] | 0.39 | 0.26 | 0.58 | -0.94 | -1.35 | -0.54 | 0.20 |
| Grotle 2006 [7] | 0.88 | 0.61 | 1.3 | -0.13 | -0.49 | 0.26 | 0.19 |
| *Meta analysis of studies within Chou* [8] | 0.50 | 0.35 | 0.71 | -0.69 | -1.04 | -0.35 | 0.18 |
| **Post-trauma stress symptoms OR** | **ES** | **L95** | **U95** | **ES ln** | **L95 ln** | **U95 ln** | **SE ln** |
| Kongsted 2008 [9] | 2.10 | 1.10 | 4.10 | 0.74 | 0.10 | 1.41 | 0.34 |
| Ravn 2019 [10] | 1.86 | 1.26 | 2.76 | 0.62 | 0.23 | 1.02 | 0.20 |
| *Meta-analysis of studies within Jadhakhan* [11] | 1.92 | 1.37 | 2.69 | 0.65 | 0.31 | 0.99 | 0.17 |
| **Poorer psychological health LR+** | **ES** | **L95** | **U95** | **ES ln** | **L95 ln** | **U95 ln** | **SE ln** |
| Turner 2006 [2] | 2.10 | 1.80 | 2.40 | 0.74 | 0.59 | 0.88 | 0.07 |
| Fransen 2002 (depression) [12] | 2.00 | 1.50 | 2.80 | 0.69 | 0.41 | 1.03 | 0.16 |
| Fransen 2002 (anxiety) [12] | 1.40 | 1.20 | 1.60 | 0.34 | 0.18 | 0.47 | 0.07 |
| Dionne 2007 (3 months) [1] | 1.90 | 1.60 | 2.40 | 0.64 | 0.47 | 0.88 | 0.10 |
| Truchon 2005 [4] | 1.60 | 1.20 | 2.00 | 0.47 | 0.18 | 0.69 | 0.13 |
| Turner 2008 [6] | 2.30 | 1.80 | 2.80 | 0.83 | 0.59 | 1.03 | 0.11 |
| Henschke 2008 (depression) [13] | 2.20 | 1.30 | 3.90 | 0.79 | 0.26 | 1.36 | 0.28 |
| Henschke 2008 (anxiety) [13] | 1.90 | 1.30 | 2.80 | 0.64 | 0.26 | 1.03 | 0.20 |
| Dionne 2007 (1 year) [1] | 2.20 | 1.80 | 2.60 | 0.79 | 0.59 | 0.96 | 0.09 |
| Cherkin 1996 [14] | 1.90 | 0.81 | 4.50 | 0.64 | -0.21 | 1.50 | 0.44 |
| *Meta-analysis of studies within Chou* [8] | 1.92 | 1.67 | 2.18 | 0.65 | 0.51 | 0.78 | 0.68 |
| **Poorer psychological health LR-** | **ES** | **L95** | **U95** | **ES ln** | **L95 ln** | **U95 ln** | **SE ln** |
| Turner 2006 [2] | 0.55 | 0.46 | 0.66 | -0.60 | -0.78 | -0.42 | 0.09 |
| Fransen 2002 (depression) [12] | 0.85 | 0.78 | 0.93 | -0.16 | -0.25 | -0.07 | 0.04 |
| Fransen 2002 (anxiety) [12] | 0.68 | 0.56 | 0.83 | -0.39 | -0.58 | -0.19 | 0.10 |
| Dionne 2007 (3 months) [1] | 0.72 | 0.63 | 0.82 | -0.33 | -0.46 | -0.20 | 0.07 |
| Truchon 2005 [4] | 0.67 | 0.53 | 0.84 | -0.40 | -0.63 | -0.17 | 0.12 |
| Turner 2008 [6] | 0.82 | 0.75 | 0.88 | -0.20 | -0.29 | -0.13 | 0.04 |
| Henschke 2008 (depression) [13] | 0.95 | 0.91 | 0.99 | -0.05 | -0.09 | -0.01 | 0.02 |
| Henschke 2008 (anxiety) [13] | 0.92 | 0.86 | 0.98 | -0.08 | -0.15 | -0.02 | 0.03 |
| Dionne 2007 (1 year) [1] | 0.65 | 0.55 | 0.76 | -0.43 | -0.60 | -0.27 | 0.08 |
| Cherkin 1996 [14] | 0.87 | 0.65 | 0.95 | -0.14 | -0.43 | -0.05 | 0.10 |
| *Meta-analysis of studies within Chou* [8] | 0.77 | 0.70 | 0.85 | -0.26 | -0.35 | -0.16 | 0.05 |
| **Catastrophising OR** | **ES** | **L95** | **U95** | **ES ln** | **L95 ln** | **U95 ln** | **SE ln** |
| *Meta-analysis by Walton (2009)* [15] | 3.99 | 1.33 | 10.74 | 1.38 | 0.29 | 2.37 | 0.53 |
| **Poorer recovery expectations OR** | **ES** | **L95** | **U95** | **ES ln** | **L95 ln** | **U95 ln** | **SE ln** |
| Kapoor 2006 [16] | 3.09 | 1.77 | 3.58 | 1.13 | 0.57 | 1.28 | 0.18 |
| Schultz 2004 [17] | 1.27 | 1.10 | 1.45 | 0.24 | 0.10 | 0.37 | 0.07 |
| Schultz 2005 [18] | 1.21 | 1.01 | 1.45 | 0.19 | 0.01 | 0.37 | 0.09 |
| Shaw 2005 [19] | 2.25 | 1.52 | 3.32 | 0.81 | 0.42 | 1.20 | 0.20 |
| Turner 2006 (very low expectations) [2] | 9.18 | 5.00 | 16.80 | 2.22 | 1.61 | 2.82 | 0.31 |
| Turner 2006 (low expectations) [2] | 6.45 | 3.38 | 12.30 | 1.86 | 1.22 | 2.51 | 0.33 |
| *Meta-analysis of studies within Iles* [20] | 2.72 | 1.68 | 4.35 | 1.00 | 0.52 | 1.47 | 0.24 |
| **Somatisation LR+** | **ES** | **L95** | **U95** | **ES ln** | **L95 ln** | **U95 ln** | **SE ln** |
| Dionne 2007 (3 months) [1] | 2.50 | 1.80 | 3.40 | 0.92 | 0.59 | 1.22 | 0.16 |
| Thomas 1999 [21] | 4.60 | 2.20 | 9.30 | 1.53 | 0.79 | 2.23 | 0.37 |
| Dionne 2007 (1 year) [1] | 3.00 | 2.20 | 4.00 | 1.10 | 0.79 | 1.39 | 0.15 |
| Turner 2008 [6] | 1.70 | 1.60 | 1.80 | 0.53 | 0.47 | 0.59 | 0.03 |
| *Meta-analysis of studies within Chou* [8] | 2.56 | 1.72 | 3.82 | 0.94 | 0.54 | 1.34 | 0.20 |
| **Somatisation LR-** | **ES** | **L95** | **U95** | **ES ln** | **L95 ln** | **U95 ln** | **SE ln** |
| Dionne 2007 (3 months) [1] | 0.81 | 0.74 | 0.89 | -0.21 | -0.30 | -0.12 | 0.05 |
| Thomas 1999 [21] | 0.71 | 0.59 | 0.86 | -0.34 | -0.53 | -0.15 | 0.10 |
| Dionne 2007 (1 year) [1] | 0.76 | 0.68 | 0.85 | -0.27 | -0.39 | -0.16 | 0.06 |
| Turner 2008 [6] | 0.31 | 0.23 | 0.41 | -1.17 | -1.47 | -0.89 | 0.15 |
| *Meta-analysis of studies within Chou* [8] | 0.63 | 0.48 | 0.82 | -0.46 | -0.73 | -0.19 | 0.14 |
| **Lower job satisfaction OR** | **ES** | **L95** | **U95** | **ES ln** | **L95 ln** | **U95 ln** | **SE ln** |
| Lang 2012 (low job satisfaction and low back pain) [22] | 1.31 | 1.02 | 1.69 | 0.27 | 0.02 | 0.52 | 0.13 |
| Lang 2012 (highly monotonous work and back pain) [22] | 1.66 | 1.34 | 2.04 | 0.51 | 0.29 | 0.71 | 0.11 |
| Lang 2012 (highly monotonous work and neck and/or shoulder symptoms) [22] | 1.30 | 1.07 | 1.57 | 0.26 | 0.07 | 0.45 | 0.10 |
| Lang 2012 (highly monotonous work and upper extremity pain) [22] | 1.62 | 1.36 | 1.97 | 0.48 | 0.31 | 0.68 | 0.09 |
| Lang 2012 (highly monotonous work and lower extremity pain) [22] | 1.15 | 0.82 | 1.62 | 0.14 | -0.20 | 0.48 | 0.17 |
| *Meta-analysis of meta-analyses within Lang* [22] | 1.43 | 1.25 | 1.63 | 0.36 | 0.23 | 0.49 | 0.67 |
| **Lower job satisfaction LR+** | **ES** | **L95** | **U95** | **ES ln** | **L95 ln** | **U95 ln** | **SE ln** |
| Dionne 2007 (3 months) [1] | 1.80 | 1.10 | 2.80 | 0.59 | 0.10 | 1.03 | 0.24 |
| Fransen 2002 [12] | 1.10 | 0.82 | 1.40 | 0.10 | -0.20 | 0.34 | 0.14 |
| Grotle 2005 [5] | 0.64 | 0.27 | 1.50 | -0.45 | -1.31 | 0.41 | 0.44 |
| Dionne 2007 (1 year) [1] | 1.80 | 1.10 | 2.90 | 0.59 | 0.10 | 1.06 | 0.25 |
| Thomas 1999 [21] | 1.50 | 1.10 | 2.90 | 0.41 | 0.10 | 1.06 | 0.25 |
| Grotle 2006 [7] | 1.30 | 0.64 | 2.80 | 0.26 | -0.45 | 1.03 | 0.38 |
| *Meta-analysis of studies within Chou* [8] | 1.35 | 1.05 | 1.74 | 0.30 | 0.05 | 0.56 | 0.13 |
| **Lower job satisfaction LR-** | **ES** | **L95** | **U95** | **ES ln** | **L95 ln** | **U95 ln** | **SE ln** |
| Dionne 2007 (3 months) [1] | 0.94 | 0.89 | 0.99 | -0.06 | -0.12 | -0.01 | 0.03 |
| Fransen 2002 [12] | 0.98 | 0.89 | 1.10 | -0.02 | -0.12 | 0.10 | 0.05 |
| Grotle 2005 [5] | 1.20 | 0.91 | 1.50 | 0.18 | -0.09 | 0.41 | 0.13 |
| Dionne 2007 (1 year) [1] | 0.94 | 0.88 | 1.00 | -0.06 | -0.13 | 0.00 | 0.03 |
| Thomas 1999 [21] | 0.62 | 0.42 | 0.91 | -0.48 | -0.87 | -0.09 | 0.20 |
| Grotle 2006 [7] | 0.88 | 0.60 | 1.30 | -0.13 | -0.51 | 0.26 | 0.20 |
| *Meta-analysis of studies within Chou* [8] | 0.95 | 0.90 | 1.01 | -0.05 | -0.11 | 0.01 | 0.03 |
| **Higher job demands OR** | **ES** | **L95** | **U95** | **ES ln** | **L95 ln** | **U95 ln** | **SE ln** |
| Lang 2012 [22] (high job demands and lower back pain) | 1.42 | 1.19 | 1.70 | 0.35 | 0.17 | 0.53 | 0.09 |
| Lang 2012 [22] (high job strain and lower back pain) | 1.38 | 1.07 | 1.78 | 0.32 | 0.07 | 0.58 | 0.13 |
| Lang 2012 [22] (high job demands and neck and/or shoulder pain) | 1.17 | 1.10 | 1.24 | 0.16 | 0.10 | 0.22 | 0.03 |
| Lang 2012 [22] (high job strain and neck and/or shoulder pain) | 1.33 | 1.08 | 1.62 | 0.29 | 0.08 | 0.48 | 0.10 |
| Lang 2012 [22] (high job demands and upper extremity symptoms) | 1.18 | 1.03 | 1.36 | 0.17 | 0.03 | 0.31 | 0.07 |
| *Meta-analysis of meta-analyses within Lang* [22] | 1.25 | 1.15 | 1.35 | 0.22 | 0.14 | 0.30 | 0.40 |
| **Higher job demands LR+** | **ES** | **L95** | **U95** | **ES ln** | **L95 ln** | **U95 ln** | **SE ln** |
| Dionne 2007 (3 months) [1] | 1.60 | 1.20 | 2.10 | 0.47 | 0.18 | 0.74 | 0.14 |
| Fransen 2002 [12] | 1.20 | 0.99 | 1.50 | 0.18 | -0.01 | 0.41 | 0.11 |
| Poiraudeau 2006 [23] | 1.10 | 0.95 | 1.30 | 0.10 | -0.05 | 0.26 | 0.08 |
| Dionne 2007 (1 year) [1] | 1.70 | 1.30 | 2.20 | 0.53 | 0.26 | 0.79 | 0.13 |
| Turner 2008 [6] | 1.20 | 1.00 | 1.30 | 0.18 | 0.00 | 0.26 | 0.07 |
| *Meta-analysis of studies within Chou* [8] | 1.30 | 1.12 | 1.51 | 0.26 | 0.11 | 0.41 | 0.77 |
| **Higher job demands LR-** | **ES** | **L95** | **U95** | **ES ln** | **L95 ln** | **U95 ln** | **SE ln** |
| Dionne 2007 (3 months) [1] | 0.87 | 0.79 | 0.96 | -0.14 | -0.24 | -0.04 | 0.05 |
| Fransen 2002 [12] | 0.89 | 0.78 | 1.00 | -0.12 | -0.25 | 0.00 | 0.06 |
| Poiraudeau 2006 [23] | 0.85 | 0.66 | 1.10 | -0.16 | -0.42 | 0.10 | 0.13 |
| Dionne 2007 (1 year) [1] | 0.85 | 0.77 | 0.95 | -0.16 | -0.26 | -0.05 | 0.05 |
| Turner 2008 [6] | 0.83 | 0.71 | 0.98 | -0.19 | -0.34 | -0.02 | 0.08 |
| *Meta-analysis of studies within Chou* [8] | 0.86 | 0.82 | 0.91 | -0.15 | -0.20 | -0.09 | 0.03 |
| **Lower job control OR** | **ES** | **L95** | **U95** | **ES ln** | **L95 ln** | **U95 ln** | **SE ln** |
| Lang 2012 (low job control and lower back pain) [22] | 1.30 | 1.11 | 1.52 | 0.26 | 0.10 | 0.42 | 0.08 |
| Lang 2012 (low job control and neck and/or shoulder pain) [22] | 1.27 | 1.17 | 1.38 | 0.24 | 0.16 | 0.32 | 0.04 |
| Lang 2012 (low job control and upper extremity pain) [22] | 1.33 | 1.11 | 1.59 | 0.29 | 0.10 | 0.46 | 0.09 |
| Lang 2012 (low job control and lower extremity pain) [22] | 1.14 | 0.74 | 1.78 | 0.13 | -0.30 | 0.58 | 0.22 |
| *Meta-analysis of meta-analyses within Lang* [22] | 1.28 | 1.20 | 1.37 | 0.25 | 0.18 | 0.31 | 0.03 |
| **Poorer support networks OR** | **ES** | **L95** | **U95** | **ES ln** | **L95 ln** | **U95 ln** | **SE ln** |
| Lang 2012 [22] (low social support and lower back pain) | 1.36 | 1.17 | 1.58 | 0.31 | 0.16 | 0.46 | 0.08 |
| Lang 2012 [22] (low supervisor support and lower back pain) | 1.33 | 1.16 | 1.53 | 0.29 | 0.15 | 0.43 | 0.07 |
| Lang 2012 [22] (low co-worker support and lower back pain) | 1.07 | 0.85 | 1.35 | 0.07 | -0.16 | 0.30 | 0.12 |
| Lang 2012 [22] (low social support and neck and/or shoulder pain) | 1.15 | 1.05 | 1.27 | 0.14 | 0.05 | 0.24 | 0.05 |
| Lang 2012 [22] (low supervisor support and neck and/or shoulder pain) | 1.17 | 1.01 | 1.34 | 0.16 | 0.01 | 0.29 | 0.07 |
| Lang 2012 [22] (low co-worker support and neck and/or shoulder pain) | 1.13 | 0.97 | 1.31 | 0.12 | -0.03 | 0.27 | 0.08 |
| Lang 2012 [22] (low social support and upper extremity pain) | 1.23 | 0.99 | 1.53 | 0.21 | -0.01 | 0.43 | 0.11 |
| Lang 2012 [22] (low social support and lower extremity pain) | 1.41 | 1.00 | 2.00 | 0.34 | 0.00 | 0.69 | 0.18 |
| *Meta-analysis of meta-analyses within Lang* [22] | 1.21 | 1.14 | 1.29 | 0.19 | 0.13 | 0.25 | 0.03 |
| **Lower socioeconomic status OR** | **ES** | **L95** | **U95** | **ES ln** | **L95 ln** | **U95 ln** | **SE ln** |
| Walton 2013 [24] | 2.00 | 1.60 | 2.51 | 0.69 | 0.47 | 0.92 | 0.11 |
| Palmlof 2012 [25] (within Buscemi 2019 [26]) | 2.00 | 1.30 | 3.20 | 0.69 | 0.26 | 1.16 | 0.23 |
| *Meta-analysis of meta-analyses* | 2.00 | 1.64 | 2.42 | 0.69 | 0.50 | 0.88 | 0.10 |
| **Lower socioeconomic status LR+** | **ES** | **L95** | **U95** | **ES ln** | **L95 ln** | **U95 ln** | **SE ln** |
| Grotle 2005 [5] | 1.30 | 0.88 | 1.90 | 0.26 | -0.13 | 0.64 | 0.20 |
| Dionne 2007 (3 months) [1] | 1.10 | 1.00 | 1.10 | 0.10 | 0.00 | 0.10 | 0.02 |
| Swinkels-Meewisse 2006 [3] | 1.10 | 1.00 | 1.30 | 0.10 | 0.00 | 0.26 | 0.07 |
| Truchon 2005 [4] | 1.00 | 0.91 | 1.20 | 0.00 | -0.09 | 0.18 | 0.07 |
| Turner 2006 [2] | 1.00 | 1.00 | 1.10 | 0.00 | 0.00 | 0.10 | 0.02 |
| Fransen 2002 [12] | 1.00 | 0.98 | 1.10 | 0.00 | -0.02 | 0.10 | 0.03 |
| Poiraudeau 2006 [23] | 0.97 | 0.86 | 1.10 | -0.03 | -0.15 | 0.10 | 0.06 |
| Grotle 2006 [7] | 1.20 | 0.76 | 2.00 | 0.18 | -0.27 | 0.69 | 0.25 |
| Dionne 2007 (1 year) [1] | 1.10 | 1.00 | 1.20 | 0.10 | 0.00 | 0.18 | 0.05 |
| Henschke 2008 [13] | 1.10 | 1.00 | 1.30 | 0.10 | 0.00 | 0.26 | 0.07 |
| Turner 2008 [6] | 1.10 | 1.00 | 1.10 | 0.10 | 0.00 | 0.10 | 0.02 |
| *Meta-analysis of studies within Chou* [8] | 1.06 | 1.02 | 1.10 | 0.06 | 0.02 | 0.10 | 0.02 |
| **Lower socioeconomic status LR-** | **ES** | **L95** | **U95** | **ES ln** | **L95 ln** | **U95 ln** | **SE ln** |
| Grotle 2005 [5] | 0.76 | 0.48 | 1.20 | -0.27 | -0.73 | 0.18 | 0.23 |
| Dionne 2007 (3 months) [1] | 0.72 | 0.49 | 1.10 | -0.33 | -0.71 | 0.10 | 0.21 |
| Swinkels-Meewisse 2006 [3] | 0.76 | 0.56 | 1.00 | -0.27 | -0.58 | 0.00 | 0.15 |
| Truchon 2005 [4] | 0.89 | 0.60 | 1.30 | -0.12 | -0.51 | 0.26 | 0.20 |
| Turner 2006 [2] | 0.52 | 0.26 | 1.00 | -0.65 | -1.35 | 0.00 | 0.34 |
| Fransen 2002 [12] | 0.74 | 0.48 | 1.20 | -0.30 | -0.73 | 0.18 | 0.23 |
| Poiraudeau 2006 [23] | 1.10 | 0.80 | 1.40 | 0.10 | -0.22 | 0.34 | 0.14 |
| Grotle 2006 [7] | 0.82 | 0.49 | 1.40 | -0.20 | -0.71 | 0.34 | 0.27 |
| Dionne 2007 (1 year) [1] | 0.51 | 0.31 | 0.85 | -0.67 | -1.17 | -0.16 | 0.26 |
| Henschke 2008 [13] | 0.85 | 0.70 | 1.00 | -0.16 | -0.36 | 0.00 | 0.09 |
| Turner 2008 [6] | 0.46 | 0.25 | 0.83 | -0.78 | -1.39 | -0.19 | 0.31 |
| *Meta-analysis of studies within Chou* [8] | 0.78 | 0.68 | 0.90 | -0.24 | -0.38 | -0.11 | 0.07 |
| **Lower job security OR** | **ES** | **L95** | **U95** | **ES ln** | **L95 ln** | **U95 ln** | **SE ln** |
| *Meta-analysis by Lang* [22] | 1.43 | 1.16 | 1.76 | 0.36 | 0.15 | 0.57 | 0.11 |
| **Financial compensation LR+** | **ES** | **L95** | **U95** | **ES ln** | **L95 ln** | **U95 ln** | **SE ln** |
| Grotle 2005 [5] | 2.70 | 0.98 | 7.40 | 0.99 | -0.02 | 2.00 | 0.52 |
| Dionne 2007 (3 months) [1] | 1.30 | 1.00 | 1.60 | 0.26 | 0.00 | 0.47 | 0.12 |
| Henschke 2008 [13] | 1.80 | 1.40 | 2.40 | 0.59 | 0.34 | 0.88 | 0.14 |
| Dixon 1999 [27] | 1.60 | 1.30 | 1.90 | 0.47 | 0.26 | 0.64 | 0.10 |
| Dionne 2007 (1 year) [1] | 1.20 | 0.90 | 1.50 | 0.18 | -0.11 | 0.41 | 0.13 |
| *Meta-analysis of studies within Chou* [8] | 1.48 | 1.24 | 1.76 | 0.39 | 0.22 | 0.57 | 0.09 |
| **Financial compensation LR-** | **ES** | **L95** | **U95** | **ES ln** | **L95 ln** | **U95 ln** | **SE ln** |
| Grotle 2005 [5] | 0.86 | 0.71 | 1.00 | -0.15 | -0.34 | 0.00 | 0.09 |
| Dionne 2007 (3 months) [1] | 0.90 | 0.80 | 1.00 | -0.11 | -0.22 | 0.00 | 0.06 |
| Henschke 2008 [13] | 0.85 | 0.78 | 0.93 | -0.16 | -0.25 | -0.07 | 0.04 |
| Dixon 1999 [27] | 0.37 | 0.18 | 0.78 | -0.99 | -1.71 | -0.25 | 0.37 |
| Dionne 2007 (1 year) [1] | 0.93 | 0.83 | 1.10 | -0.07 | -0.19 | 0.10 | 0.07 |
| *Meta-analysis of studies within Chou* [8] | 0.87 | 0.80 | 0.95 | -0.14 | -0.22 | -0.05 | 0.04 |
| **High levels of pain at or near onset OR** | **ES** | **L95** | **U95** | **ES ln** | **L95 ln** | **U95 ln** | **SE ln** |
| *Meta-analysis by Walton (2013)* [24] | 5.61 | 3.74 | 8.43 | 1.73 | 1.32 | 2.13 | 0.21 |
| **High levels of pain at or near onset LR+** | **ES** | **L95** | **U95** | **ES ln** | **L95 ln** | **U95 ln** | **SE ln** |
| Singer 1987 (3 months) [28] | 3.70 | 1.90 | 7.40 | 1.31 | 0.64 | 2.00 | 0.35 |
| Poiraudeau 2006 [23] | 3.40 | 1.40 | 8.80 | 1.22 | 0.34 | 2.17 | 0.47 |
| Dionne 2007 (3 months) [1] | 2.00 | 1.70 | 2.40 | 0.69 | 0.53 | 0.88 | 0.09 |
| Turner 2006 [2] | 2.00 | 1.80 | 2.40 | 0.69 | 0.59 | 0.88 | 0.07 |
| Truchon 2005 [4] | 1.50 | 1.20 | 1.80 | 0.41 | 0.18 | 0.59 | 0.10 |
| Swinkels-Meewisse 2006 [3] | 1.20 | 0.88 | 1.70 | 0.18 | -0.13 | 0.53 | 0.17 |
| Fransen 2002 [12] | 1.10 | 1.00 | 1.20 | 0.10 | 0.00 | 0.18 | 0.05 |
| Dionne 2007 (1 year) [1] | 2.00 | 1.70 | 2.40 | 0.69 | 0.53 | 0.88 | 0.09 |
| Turner 2008 [6] | 2.00 | 1.80 | 2.30 | 0.69 | 0.59 | 0.83 | 0.06 |
| Henschke 2008 [13] | 1.30 | 1.20 | 1.50 | 0.26 | 0.18 | 0.41 | 0.06 |
| Singer 1987 (1 year) [28] | 1.20 | 0.49 | 2.80 | 0.18 | -0.71 | 1.03 | 0.44 |
| *Meta-analysis of studies within Chou* [8] | 1.69 | 1.39 | 2.04 | 0.52 | 0.33 | 0.72 | 0.10 |
| **High levels of pain at or near onset LR-** | **ES** | **L95** | **U95** | **ES ln** | **L95 ln** | **U95 ln** | **SE ln** |
| Singer 1987 (3 months) [28] | 0.71 | 0.47 | 1.10 | -0.34 | -0.76 | 0.10 | 0.22 |
| Poiraudeau 2006 [23] | 0.70 | 0.56 | 0.87 | -0.36 | -0.58 | -0.14 | 0.11 |
| Dionne 2007 (3 months) [1] | 0.56 | 0.47 | 0.67 | -0.58 | -0.76 | -0.40 | 0.09 |
| Turner 2006 [2] | 0.07 | 0.03 | 0.18 | -2.66 | -3.51 | -1.71 | 0.46 |
| Truchon 2005 [4] | 0.54 | 0.23 | 1.20 | -0.62 | -1.47 | 0.18 | 0.42 |
| Swinkels-Meewisse 2006 [3] | 0.86 | 0.66 | 1.10 | -0.15 | -0.42 | 0.10 | 0.13 |
| Fransen 2002 [12] | 0.76 | 0.40 | 1.40 | -0.27 | -0.92 | 0.34 | 0.32 |
| Dionne 2007 (1 year) [1] | 0.54 | 0.44 | 0.66 | -0.62 | -0.82 | -0.42 | 0.10 |
| Turner 2008 [6] | 0.08 | 0.04 | 0.18 | -2.53 | -3.22 | -1.71 | 0.38 |
| Henschke 2008 [13] | 0.33 | 0.08 | 1.40 | -1.11 | -2.53 | 0.34 | 0.73 |
| Singer 1987 (1 year) [28] | 0.97 | 0.73 | 1.30 | -0.03 | -0.31 | 0.26 | 0.15 |
| *Meta-analysis of studies within Chou* [8] | 0.51 | 0.38 | 0.68 | -0.67 | -0.96 | -0.38 | 0.15 |
| **Concomitant pain OR** | **ES** | **L95** | **U95** | **ES ln** | **L95 ln** | **U95 ln** | **SE ln** |
| *Meta-analysis by Walton (2013)* [24] | 1.83 | 1.25 | 2.67 | 0.60 | 0.22 | 0.98 | 0.19 |
| **Disturbed sleep since onset OR** | **ES** | **L95** | **U95** | **ES ln** | **L95 ln** | **U95 ln** | **SE ln** |
| *Meta-analysis by Walton (2009)* [15] | 2.96 | 0.97 | 9.04 | 1.09 | -0.03 | 2.20 | 0.57 |
| **Higher levels of functional impairment at onset LR+** | **ES** | **L95** | **U95** | **ES ln** | **L95 ln** | **U95 ln** | **SE ln** |
| Dionne 2007 (3 months) [1] | 3.50 | 2.70 | 4.60 | 1.25 | 0.99 | 1.53 | 0.14 |
| Turner 2006 [2] | 3.00 | 2.50 | 3.60 | 1.10 | 0.92 | 1.28 | 0.09 |
| Fransen 2002 [12] | 1.40 | 1.10 | 1.70 | 0.34 | 0.10 | 0.53 | 0.11 |
| Poiraudeau 2006 [23] | 1.40 | 1.10 | 1.60 | 0.34 | 0.10 | 0.47 | 0.10 |
| Swinkels-Meewisse 2006 [3] | 1.40 | 0.80 | 2.60 | 0.34 | -0.22 | 0.96 | 0.30 |
| Truchon 2005 [4] | 1.30 | 0.84 | 2.00 | 0.26 | -0.17 | 0.69 | 0.22 |
| Turner 2008 [6] | 2.70 | 2.40 | 3.00 | 0.99 | 0.88 | 1.10 | 0.06 |
| Dionne 2007 (1 year) [1] | 2.10 | 1.60 | 2.80 | 0.74 | 0.47 | 1.03 | 0.14 |
| Henschke 2008 [13] | 1.20 | 1.00 | 1.30 | 0.18 | 0.00 | 0.26 | 0.07 |
| *Meta-analysis of studies within Chou* [8] | 1.88 | 1.40 | 2.51 | 0.63 | 0.34 | 0.92 | 0.15 |
| **Higher levels of functional impairment at onset LR-** | **ES** | **L95** | **U95** | **ES ln** | **L95 ln** | **U95 ln** | **SE ln** |
| Dionne 2007 (3 months) [1] | 0.33 | 0.25 | 0.44 | -1.11 | -1.39 | -0.82 | 0.14 |
| Turner 2006 [2] | 0.18 | 0.12 | 0.28 | -1.71 | -2.12 | -1.27 | 0.22 |
| Fransen 2002 [12] | 0.59 | 0.47 | 0.74 | -0.53 | -0.76 | -0.30 | 0.12 |
| Poiraudeau 2006 [23] | 0.52 | 0.22 | 1.20 | -0.65 | -1.51 | 0.18 | 0.43 |
| Swinkels-Meewisse 2006 [3] | 1.10 | 0.82 | 1.40 | 0.10 | -0.20 | 0.34 | 0.14 |
| Truchon 2005 [4] | 0.54 | 0.38 | 0.75 | -0.62 | -0.97 | -0.29 | 0.17 |
| Turner 2008 [6] | 0.10 | 0.06 | 0.18 | -2.30 | -2.81 | -1.71 | 0.28 |
| Dionne 2007 (1 year) [1] | 0.40 | 0.30 | 0.54 | -0.92 | -1.20 | -0.62 | 0.15 |
| Henschke 2008 [13] | 0.52 | 0.25 | 1.10 | -0.65 | -1.39 | 0.10 | 0.38 |
| *Meta-analysis of studies within Chou* [8] | 0.40 | 0.26 | 0.61 | -0.92 | -1.35 | -0.50 | 0.22 |
| **Cold hyperalgesia OR** | **ES** | **L95** | **U95** | **ES ln** | **L95 ln** | **U95 ln** | **SE ln** |
| Sterling 2005 [29] | 1.29 | 1.05 | 1.58 | 0.25 | 0.05 | 0.46 | 0.10 |
| Sterling 2006 [30] | 26.32 | 4.98 | 139.09 | 3.27 | 1.61 | 4.94 | 0.85 |
| Sterling 2011 [31] | 1.05 | 1.00 | 1.13 | 0.05 | 0.00 | 0.12 | 0.03 |
| *Meta-analysis of studies within Goldsmith* [32] | 1.36 | 0.91 | 2.05 | 0.31 | -0.10 | 0.72 | 0.21 |
| **Female gender OR** | **ES** | **L95** | **U95** | **ES ln** | **L95 ln** | **U95 ln** | **SE ln** |
| Agnello 2010 [33] | 1.28 | 1.03 | 1.58 | 0.25 | 0.03 | 0.46 | 0.11 |
| Walton 2013 [24] | 1.64 | 1.27 | 2.12 | 0.49 | 0.24 | 0.75 | 0.13 |
| *Meta-analysis of meta-analyses* | 1.43 | 1.13 | 1.81 | 0.36 | 0.13 | 0.59 | 0.12 |
| **Female sex LR+** | **ES** | **L95** | **U95** | **ES ln** | **L95 ln** | **U95 ln** | **SE ln** |
| Poiraudeau 2006 [23] | 1.40 | 1.10 | 1.70 | 0.34 | 0.10 | 0.53 | 0.11 |
| Grotle 2005 [5] | 1.30 | 0.96 | 1.80 | 0.26 | -0.04 | 0.59 | 0.16 |
| Heneweer 2007 [34] | 1.20 | 0.65 | 2.40 | 0.18 | -0.43 | 0.88 | 0.33 |
| Fransen 2002 [12] | 1.10 | 0.83 | 1.40 | 0.10 | -0.19 | 0.34 | 0.13 |
| Dionne 2007 (3 months) [1] | 1.10 | 0.92 | 1.30 | 0.10 | -0.08 | 0.26 | 0.09 |
| Schiottz-Christensen 1999 (6 months) [35] | 1.10 | 0.85 | 1.54 | 0.10 | -0.16 | 0.43 | 0.15 |
| Swinkels-Meewisse 2006 [3] | 1.00 | 0.81 | 1.30 | 0.00 | -0.21 | 0.26 | 0.12 |
| Turner 2006 [2] | 0.92 | 0.72 | 1.20 | -0.08 | -0.33 | 0.18 | 0.13 |
| Truchon 2005 [4] | 0.72 | 0.55 | 0.94 | -0.33 | -0.60 | -0.06 | 0.14 |
| Dixon 1999 [27] | 1.70 | 1.30 | 2.30 | 0.53 | 0.26 | 0.83 | 0.15 |
| Cherkin 1996 [14] | 1.50 | 1.20 | 2.00 | 0.41 | 0.18 | 0.69 | 0.13 |
| Henschke 2008 [13] | 1.30 | 1.10 | 1.50 | 0.26 | 0.10 | 0.41 | 0.08 |
| Grotle 2006 [7] | 1.30 | 0.88 | 1.80 | 0.26 | -0.13 | 0.59 | 0.18 |
| Thomas 1999 [21] | 1.30 | 1.00 | 1.70 | 0.26 | 0.00 | 0.53 | 0.14 |
| Dionne 2007 (1 year) [1] | 1.00 | 0.85 | 1.30 | 0.00 | -0.16 | 0.26 | 0.11 |
| Schiottz-Christensen 1999 (1 year) [35] | 1.00 | 0.78 | 1.40 | 0.00 | -0.25 | 0.34 | 0.15 |
| Turner 2008 [6] | 1.00 | 0.83 | 1.20 | 0.00 | -0.19 | 0.18 | 0.09 |
| *Meta-analysis of studies within Chou* [8] | 1.14 | 1.04 | 1.26 | 0.14 | 0.04 | 0.23 | 0.05 |
| **Female sex LR-** | **ES** | **L95** | **U95** | **ES ln** | **L95 ln** | **U95 ln** | **SE ln** |
| Poiraudeau 2006 [23] | 0.78 | 0.66 | 0.93 | -0.25 | -0.42 | -0.07 | 0.09 |
| Grotle 2005 [5] | 0.66 | 0.37 | 1.20 | -0.42 | -0.99 | 0.18 | 0.30 |
| Heneweer 2007 [34] | 0.87 | 0.56 | 1.30 | -0.14 | -0.58 | 0.26 | 0.21 |
| Fransen 2002 [12] | 0.98 | 0.89 | 1.10 | -0.02 | -0.12 | 0.10 | 0.05 |
| Dionne 2007 (3 months) [1] | 0.93 | 0.80 | 1.10 | -0.07 | -0.22 | 0.10 | 0.08 |
| Schiottz-Christensen 1999 (6 months) [35] | 0.94 | 0.78 | 1.10 | -0.06 | -0.25 | 0.10 | 0.09 |
| Swinkels-Meewisse 2006 [3] | 0.99 | 0.83 | 1.20 | -0.01 | -0.19 | 0.18 | 0.09 |
| Turner 2006 [2] | 1.00 | 0.94 | 1.20 | 0.00 | -0.06 | 0.18 | 0.06 |
| Truchon 2005 [4] | 1.30 | 1.10 | 1.50 | 0.26 | 0.10 | 0.41 | 0.08 |
| Dixon 1999 [27] | 0.58 | 0.38 | 0.89 | -0.54 | -0.97 | -0.12 | 0.22 |
| Cherkin 1996 [14] | 0.62 | 0.43 | 0.90 | -0.48 | -0.84 | -0.11 | 0.19 |
| Henschke 2008 [13] | 0.79 | 0.67 | 0.93 | -0.24 | -0.40 | -0.07 | 0.08 |
| Grotle 2006 [7] | 0.68 | 0.34 | 1.40 | -0.39 | -1.08 | 0.34 | 0.36 |
| Thomas 1999 [21] | 0.63 | 0.41 | 0.97 | -0.46 | -0.89 | -0.03 | 0.22 |
| Dionne 2007 (1 year) [1] | 0.97 | 0.84 | 1.10 | -0.03 | -0.17 | 0.10 | 0.07 |
| Schiottz-Christensen 1999 (1 year) [35] | 0.98 | 0.81 | 1.20 | -0.02 | -0.21 | 0.18 | 0.10 |
| Turner 2008 [6] | 1.00 | 0.91 | 1.10 | 0.00 | -0.09 | 0.10 | 0.05 |
| *Meta-analysis of studies within Chou* [8] | 0.92 | 0.85 | 0.99 | -0.09 | -0.16 | -0.01 | 0.04 |
| **Higher age OR** | **ES** | **L95** | **U95** | **ES ln** | **L95 ln** | **U95 ln** | **SE ln** |
| *Meta-analysis by Walton (2013)* [24] | 1.00 | 0.97 | 1.04 | 0.00 | -0.03 | 0.04 | 0.02 |
| **Higher age LR+** | **ES** | **L95** | **U95** | **ES ln** | **L95 ln** | **U95 ln** | **SE ln** |
| Schiottz-Christensen 1999 (3 months) [35] | 0.92 | 0.73 | 1.20 | -0.08 | -0.31 | 0.18 | 0.13 |
| Truchon 2005 [4] | 0.81 | 0.58 | 1.10 | -0.21 | -0.54 | 0.10 | 0.16 |
| Dionne 2007 (3 months) [1] | 1.10 | 0.87 | 1.40 | 0.10 | -0.14 | 0.34 | 0.12 |
| Fransen 2002 [12] | 1.20 | 0.93 | 1.50 | 0.18 | -0.07 | 0.41 | 0.12 |
| Turner 2006 [2] | 1.30 | 1.00 | 1.60 | 0.26 | 0.00 | 0.47 | 0.12 |
| Grotle 2005 [5] | 2.00 | 1.10 | 3.50 | 0.69 | 0.10 | 1.25 | 0.30 |
| Schiottz-Christensen 1999 (1 year) [35] | 0.99 | 0.79 | 1.20 | -0.01 | -0.24 | 0.18 | 0.11 |
| Dionne 2007 (1 year) [1] | 1.00 | 0.78 | 1.30 | 0.00 | -0.25 | 0.26 | 0.13 |
| Henschke 2008 [13] | 1.00 | 0.89 | 1.20 | 0.00 | -0.12 | 0.18 | 0.08 |
| Turner 2008 [6] | 1.20 | 1.00 | 1.40 | 0.18 | 0.00 | 0.34 | 0.09 |
| Grotle 2006 [7] | 1.80 | 0.94 | 3.40 | 0.59 | -0.06 | 1.22 | 0.33 |
| Thomas 1999 [21] | 1.40 | 1.10 | 1.70 | 0.34 | 0.10 | 0.53 | 0.11 |
| *Meta-analysis of studies within Chou* [8] | 1.12 | 1.01 | 1.24 | 0.11 | 0.01 | 0.22 | 0.05 |
| **Higher age LR-** | **ES** | **L95** | **U95** | **ES ln** | **L95 ln** | **U95 ln** | **SE ln** |
| Schiottz-Christensen 1999 (3 months) [35] | 1.10 | 0.88 | 1.40 | 0.10 | -0.13 | 0.34 | 0.12 |
| Truchon 2005 [4] | 1.10 | 0.95 | 1.30 | 0.10 | -0.05 | 0.26 | 0.08 |
| Dionne 2007 (3 months) [1] | 0.96 | 0.86 | 1.10 | -0.04 | -0.15 | 0.10 | 0.06 |
| Fransen 2002 [12] | 0.93 | 0.84 | 1.00 | -0.07 | -0.17 | 0.00 | 0.04 |
| Turner 2006 [2] | 0.88 | 0.78 | 0.99 | -0.13 | -0.25 | -0.01 | 0.06 |
| Grotle 2005 [5] | 0.74 | 0.54 | 1.00 | -0.30 | -0.62 | 0.00 | 0.16 |
| Schiottz-Christensen 1999 (1 year) [35] | 1.00 | 0.79 | 1.30 | 0.00 | -0.24 | 0.26 | 0.13 |
| Dionne 2007 (1 year) [1] | 1.00 | 0.89 | 1.10 | 0.00 | -0.12 | 0.10 | 0.05 |
| Henschke 2008 [13] | 0.97 | 0.84 | 1.10 | -0.03 | -0.17 | 0.10 | 0.07 |
| Turner 2008 [6] | 0.90 | 0.81 | 1.00 | -0.11 | -0.21 | 0.00 | 0.05 |
| Grotle 2006 [7] | 0.76 | 0.51 | 1.10 | -0.27 | -0.67 | 0.10 | 0.20 |
| Thomas 1999 [21] | 0.62 | 0.40 | 0.95 | -0.48 | -0.92 | -0.05 | 0.22 |
| *Meta-analysis of studies within Chou* [8] | 0.94 | 0.89 | 1.00 | -0.06 | -0.11 | 0.00 | 0.03 |
| **Poorer general health LR+** | **ES** | **L95** | **U95** | **ES ln** | **L95 ln** | **U95 ln** | **SE ln** |
| Fransen 2002 [12] | 1.70 | 1.50 | 2.10 | 0.53 | 0.41 | 0.74 | 0.09 |
| Dionne 2007 (3 months) [1] | 1.60 | 1.20 | 2.20 | 0.47 | 0.18 | 0.79 | 0.15 |
| Poiraudeau 2006 [23] | 1.10 | 0.99 | 1.20 | 0.10 | -0.01 | 0.18 | 0.05 |
| Thomas 1999 (self-rated poor-excellent) [21] | 2.00 | 1.40 | 2.80 | 0.69 | 0.34 | 1.03 | 0.18 |
| Thomas 1999 (SF36 general health) [21] | 1.40 | 1.20 | 1.70 | 0.34 | 0.18 | 0.53 | 0.09 |
| Cherkin 1996 [14] | 1.80 | 1.10 | 2.80 | 0.59 | 0.10 | 1.03 | 0.24 |
| Dionne 2007 (1 year) [1] | 1.80 | 1.40 | 2.50 | 0.59 | 0.34 | 0.92 | 0.15 |
| Henschke 2008 [13] | 1.60 | 0.95 | 2.60 | 0.47 | -0.05 | 0.96 | 0.26 |
| Turner 2008 [6] | 1.10 | 0.74 | 1.60 | 0.10 | -0.30 | 0.47 | 0.20 |
| *Meta-analysis of studies within Chou* [8] | 1.51 | 1.27 | 1.80 | 0.41 | 0.24 | 0.59 | 0.88 |
| **Poorer general health LR-** | **ES** | **L95** | **U95** | **ES ln** | **L95 ln** | **U95 ln** | **SE ln** |
| Fransen 2002 [12] | 0.66 | 0.56 | 0.78 | -0.42 | -0.58 | -0.25 | 0.08 |
| Dionne 2007 (3 months) [1] | 0.88 | 0.81 | 0.96 | -0.13 | -0.21 | -0.04 | 0.04 |
| Poiraudeau 2006 [23] | 0.73 | 0.51 | 1.00 | -0.31 | -0.67 | 0.00 | 0.17 |
| Thomas 1999 (self-rated poor-excellent) [21] | 0.56 | 0.40 | 0.79 | -0.58 | -0.92 | -0.24 | 0.17 |
| Thomas 1999 (SF36 general health) [21] | 0.42 | 0.23 | 0.77 | -0.87 | -1.47 | -0.26 | 0.31 |
| Cherkin 1996 [14] | 0.79 | 0.63 | 0.99 | -0.24 | -0.46 | -0.01 | 0.12 |
| Dionne 2007 (1 year) [1] | 0.85 | 0.77 | 0.94 | -0.16 | -0.26 | -0.06 | 0.05 |
| Henschke 2008 [13] | 0.96 | 0.92 | 1.00 | -0.04 | -0.08 | 0.00 | 0.02 |
| Turner 2008 [6] | 0.99 | 0.95 | 1.00 | -0.01 | -0.05 | 0.00 | 0.01 |
| *Meta-analysis of studies within Chou* [8] | 0.84 | 0.77 | 0.91 | -0.18 | -0.26 | -0.10 | 0.04 |
| **High BMI OR** | **ES** | **L95** | **U95** | **ES ln** | **L95 ln** | **U95 ln** | **SE ln** |
| *Meta-analysis by Walton (2009)* [15] | 1.24 | 0.71 | 2.19 | 0.22 | -0.34 | 0.78 | 0.29 |
| **High BMI LR+** | **ES** | **L95** | **U95** | **ES ln** | **L95 ln** | **U95 ln** | **SE ln** |
| Fransen 2002 [12] | 1.20 | 1.10 | 1.40 | 0.18 | 0.10 | 0.34 | 0.06 |
| Dionne 2007 (3 months) [1] | 0.91 | 0.71 | 1.20 | -0.09 | -0.34 | 0.18 | 0.13 |
| Schiottz-Christensen 1999 (3 months) [35] | 0.72 | 0.53 | 0.98 | -0.33 | -0.63 | -0.02 | 0.16 |
| Dionne 2007 (1 year) [1] | 0.97 | 0.74 | 1.30 | -0.03 | -0.30 | 0.26 | 0.14 |
| Schiottz-Christensen 1999 (1 year) [35] | 0.73 | 0.53 | 1.00 | -0.31 | -0.63 | 0.00 | 0.16 |
| *Meta-analysis of studies within Chou* [8] | 0.91 | 0.73 | 1.14 | -0.09 | -0.31 | 0.13 | 0.11 |
| **High BMI LR-** | **ES** | **L95** | **U95** | **ES ln** | **L95 ln** | **U95 ln** | **SE ln** |
| Fransen 2002 [12] | 0.76 | 0.63 | 0.92 | -0.27 | -0.46 | -0.08 | 0.10 |
| Dionne 2007 (3 months) [1] | 1.00 | 0.94 | 1.20 | 0.00 | -0.06 | 0.18 | 0.06 |
| Schiottz-Christensen 1999 (3 months) [35] | 1.20 | 1.00 | 1.40 | 0.18 | 0.00 | 0.34 | 0.09 |
| Dionne 2007 (1 year) [1] | 1.00 | 0.90 | 1.10 | 0.00 | -0.11 | 0.10 | 0.05 |
| Schiottz-Christensen 1999 (1 year) [35] | 1.20 | 1.00 | 1.40 | 0.18 | 0.00 | 0.34 | 0.09 |
| *Meta-analysis of studies within Chou* [8] | 1.02 | 0.90 | 1.16 | 0.02 | -0.11 | 0.15 | 0.06 |
| **Smoking OR** | **ES** | **L95** | **U95** | **ES ln** | **L95 ln** | **U95 ln** | **SE ln** |
| Van Oostrom 2011 [36] | 1.27 | 1.04 | 1.56 | 0.24 | 0.04 | 0.44 | 0.10 |
| Hussain 2016 [37] | 1.41 | 1.11 | 1.79 | 0.34 | 0.10 | 0.58 | 0.12 |
| Kaaria 2012 [38] | 1.06 | 0.87 | 1.29 | 0.06 | -0.14 | 0.25 | 0.10 |
| Kvalheim 2013 [39] | 1.24 | 1.15 | 1.34 | 0.22 | 0.14 | 0.29 | 0.04 |
| *Meta-analysis of studies within Dai* [40] | 1.24 | 1.14 | 1.34 | 0.21 | 0.13 | 0.29 | 0.04 |
| **Smoking LR+** | **ES** | **L95** | **U95** | **ES ln** | **L95 ln** | **U95 ln** | **SE ln** |
| Grotle 2005 [5] | 1.60 | 1.00 | 2.60 | 0.47 | 0.00 | 0.96 | 0.24 |
| Dionne 2007 (3 months) [1] | 1.20 | 1.00 | 1.40 | 0.18 | 0.00 | 0.34 | 0.09 |
| Fransen 2002 [12] | 1.00 | 0.86 | 1.30 | 0.00 | -0.15 | 0.26 | 0.11 |
| Grotle 2006 [7] | 1.40 | 0.80 | 2.40 | 0.34 | -0.22 | 0.88 | 0.28 |
| Henschke 2008 [13] | 1.40 | 1.10 | 1.90 | 0.34 | 0.10 | 0.64 | 0.14 |
| Thomas 1999 [21] | 1.20 | 1.00 | 1.40 | 0.18 | 0.00 | 0.34 | 0.09 |
| Dionne 2007 (1 year) [1] | 1.10 | 0.92 | 1.40 | 0.10 | -0.08 | 0.34 | 0.11 |
| *Meta-analysis of studies within Chou* [8] | 1.18 | 1.08 | 1.30 | 0.17 | 0.08 | 0.26 | 0.05 |
| **Smoking LR-** | **ES** | **L95** | **U95** | **ES ln** | **L95 ln** | **U95 ln** | **SE ln** |
| Grotle 2005 [5] | 0.71 | 0.47 | 1.10 | -0.34 | -0.76 | 0.10 | 0.22 |
| Dionne 2007 (3 months) [1] | 0.88 | 0.77 | 1.00 | -0.13 | -0.26 | 0.00 | 0.07 |
| Fransen 2002 [12] | 0.97 | 0.85 | 1.10 | -0.03 | -0.16 | 0.10 | 0.07 |
| Grotle 2006 [7] | 0.80 | 0.51 | 1.20 | -0.22 | -0.67 | 0.18 | 0.22 |
| Henschke 2008 [13] | 0.91 | 0.84 | 0.98 | -0.09 | -0.17 | -0.02 | 0.04 |
| Thomas 1999 [21] | 0.58 | 0.33 | 1.00 | -0.54 | -1.11 | 0.00 | 0.28 |
| Dionne 2007 (1 year) [1] | 0.92 | 0.80 | 1.10 | -0.08 | -0.22 | 0.10 | 0.08 |
| *Meta-analysis of studies within Chou* [8] | 0.91 | 0.86 | 0.96 | -0.10 | -0.15 | -0.04 | 0.03 |
| **History of the same MSK pain OR** | **ES** | **L95** | **U95** | **ES ln** | **L95 ln** | **U95 ln** | **SE ln** |
| Agnello 2010 [33] | 0.91 | 0.52 | 1.60 | -0.09 | -0.65 | 0.47 | 0.29 |
| Walton 2009 [15] | 1.59 | 1.03 | 2.46 | 0.46 | 0.03 | 0.90 | 0.22 |
| *Meta-analysis of meta-analyses* | 1.24 | 0.73 | 2.12 | 0.22 | -0.32 | 0.75 | 0.27 |
| **History of the same MSK pain LR+** | **ES** | **L95** | **U95** | **ES ln** | **L95 ln** | **U95 ln** | **SE ln** |
| Swinkels-Meewisse 2006 [3] | 1.20 | 1.10 | 1.30 | 0.18 | 0.10 | 0.26 | 0.04 |
| Heneweer 2007 [34] | 1.10 | 0.64 | 1.80 | 0.10 | -0.45 | 0.59 | 0.26 |
| Singer 1987 (3 months) [28] | 1.10 | 0.89 | 1.40 | 0.10 | -0.12 | 0.34 | 0.12 |
| Dionne 2007 (3 months) [1] | 1.00 | 0.97 | 1.10 | 0.00 | -0.03 | 0.10 | 0.03 |
| Grotle 2005 [5] | 0.94 | 0.66 | 1.40 | -0.06 | -0.42 | 0.34 | 0.19 |
| Fransen 2002 [12] | 0.90 | 0.72 | 1.10 | -0.11 | -0.33 | 0.10 | 0.11 |
| Thomas 1999 [21] | 1.20 | 1.00 | 1.30 | 0.18 | 0.00 | 0.26 | 0.07 |
| Dionne 2007 (1 year) [1] | 1.10 | 1.00 | 1.20 | 0.10 | 0.00 | 0.18 | 0.05 |
| Henschke 2008 [13] | 1.10 | 0.98 | 1.20 | 0.10 | -0.02 | 0.18 | 0.05 |
| Singer 1987 (1 year) [28] | 1.00 | 0.84 | 1.30 | 0.00 | -0.17 | 0.26 | 0.11 |
| Grotle 2006 [7] | 0.95 | 0.62 | 1.40 | -0.05 | -0.48 | 0.34 | 0.21 |
| *Meta-analysis of studies within Chou* [8] | 1.08 | 1.02 | 1.15 | 0.08 | 0.02 | 0.14 | 0.03 |
| **History of the same MSK pain LR-** | **ES** | **L95** | **U95** | **ES ln** | **L95 ln** | **U95 ln** | **SE ln** |
| Swinkels-Meewisse 2006 [3] | 0.53 | 0.34 | 0.83 | -0.63 | -1.08 | -0.19 | 0.23 |
| Heneweer 2007 [34] | 0.93 | 0.55 | 1.60 | -0.07 | -0.60 | 0.47 | 0.27 |
| Singer 1987 (3 months) [28] | 0.78 | 0.41 | 1.50 | -0.25 | -0.89 | 0.41 | 0.33 |
| Dionne 2007 (3 months) [1] | 0.84 | 0.61 | 1.20 | -0.17 | -0.49 | 0.18 | 0.17 |
| Grotle 2005 [5] | 1.10 | 0.66 | 1.80 | 0.10 | -0.42 | 0.59 | 0.26 |
| Fransen 2002 [12] | 1.00 | 0.94 | 1.20 | 0.00 | -0.06 | 0.18 | 0.06 |
| Thomas 1999 [21] | 0.32 | 0.12 | 0.90 | -1.14 | -2.12 | -0.11 | 0.51 |
| Dionne 2007 (1 year) [1] | 0.66 | 0.45 | 0.98 | -0.42 | -0.80 | -0.02 | 0.20 |
| Henschke 2008 [13] | 0.81 | 0.61 | 1.10 | -0.21 | -0.49 | 0.10 | 0.15 |
| Singer 1987 (1 year) [28] | 0.93 | 0.57 | 1.50 | -0.07 | -0.56 | 0.41 | 0.25 |
| Grotle 2006 [7] | 1.10 | 0.60 | 2.00 | 0.10 | -0.51 | 0.69 | 0.31 |
| *Meta-analysis of studies within Chou* [8] | 0.84 | 0.72 | 0.98 | -0.18 | -0.33 | -0.02 | 0.08 |

1. Dionne CE, Bourbonnais R, Frémont P, Rossignol M, Stock SR, Nouwen A, et al. Determinants of "return to work in good health" among workers with back pain who consult in primary care settings: a 2-year prospective study. Eur Spine J. 2007;16(5):641-55. Epub 2006/07/27. doi: 10.1007/s00586-006-0180-2. PubMed PMID: 16868783; PubMed Central PMCID: PMCPMC2213556.

2. Turner JA, Franklin G, Fulton-Kehoe D, Sheppard L, Wickizer TM, Wu R, et al. Worker recovery expectations and fear-avoidance predict work disability in a population-based workers' compensation back pain sample. Spine (Phila Pa 1976). 2006;31(6):682-9. Epub 2006/03/17. doi: 10.1097/01.brs.0000202762.88787.af. PubMed PMID: 16540874.

3. Swinkels-Meewisse IE, Roelofs J, Schouten EG, Verbeek AL, Oostendorp RA, Vlaeyen JW. Fear of movement/(re)injury predicting chronic disabling low back pain: a prospective inception cohort study. Spine (Phila Pa 1976). 2006;31(6):658-64. Epub 2006/03/17. doi: 10.1097/01.brs.0000203709.65384.9d. PubMed PMID: 16540870.

4. Truchon M, Côté D. Predictive validity of the Chronic Pain Coping Inventory in subacute low back pain. Pain. 2005;116(3):205-12. Epub 2005/06/02. doi: 10.1016/j.pain.2005.04.003. PubMed PMID: 15927382.

5. Grotle M, Brox JI, Veierød MB, Glomsrød B, Lønn JH, Vøllestad NK. Clinical course and prognostic factors in acute low back pain: patients consulting primary care for the first time. Spine (Phila Pa 1976). 2005;30(8):976-82. Epub 2005/04/19. doi: 10.1097/01.brs.0000158972.34102.6f. PubMed PMID: 15834343.

6. Turner JA, Franklin G, Fulton-Kehoe D, Sheppard L, Stover B, Wu R, et al. ISSLS prize winner: early predictors of chronic work disability: a prospective, population-based study of workers with back injuries. Spine (Phila Pa 1976). 2008;33(25):2809-18. Epub 2008/12/04. doi: 10.1097/BRS.0b013e31817df7a7. PubMed PMID: 19050587.

7. Grotle M, Vøllestad NK, Brox JI. Clinical course and impact of fear-avoidance beliefs in low back pain: prospective cohort study of acute and chronic low back pain: II. Spine (Phila Pa 1976). 2006;31(9):1038-46. Epub 2006/04/28. doi: 10.1097/01.brs.0000214878.01709.0e. PubMed PMID: 16641782.

8. Chou R, Shekelle P. Will this patient develop persistent disabling low back pain? Jama. 2010;303(13):1295-302. Epub 2010/04/08. doi: 10.1001/jama.2010.344. PubMed PMID: 20371789.

9. Kongsted A, Bendix T, Qerama E, Kasch H, Bach FW, Korsholm L, et al. Acute stress response and recovery after whiplash injuries. A one-year prospective study. European Journal of Pain. 2008;12(4):455-63. doi: <https://doi.org/10.1016/j.ejpain.2007.07.008>.

10. Ravn SL, Karstoft K-I, Sterling M, Andersen TE. Trajectories of posttraumatic stress symptoms after whiplash: A prospective cohort study. European Journal of Pain. 2019;23(3):515-25. doi: <https://doi.org/10.1002/ejp.1325>.

11. Jadhakhan F, Evans DW, Falla D. The role of post-trauma stress symptoms in the development of chronic musculoskeletal pain and disability: A systematic review. Eur J Pain. 2023;27(2):183-200. Epub 2022/11/02. doi: 10.1002/ejp.2048. PubMed PMID: 36317593.

12. Fransen M, Woodward M, Norton R, Coggan C, Dawe M, Sheridan N. Risk factors associated with the transition from acute to chronic occupational back pain. Spine (Phila Pa 1976). 2002;27(1):92-8. Epub 2002/01/24. doi: 10.1097/00007632-200201010-00022. PubMed PMID: 11805644.

13. Henschke N, Maher CG, Refshauge KM, Herbert RD, Cumming RG, Bleasel J, et al. Prognosis in patients with recent onset low back pain in Australian primary care: inception cohort study. Bmj. 2008;337(7662):a171. Epub 2008/07/11. doi: 10.1136/bmj.a171. PubMed PMID: 18614473; PubMed Central PMCID: PMCPMC2483884.

14. Cherkin DC, Deyo RA, Street JH, Barlow W. Predicting poor outcomes for back pain seen in primary care using patients' own criteria. Spine (Phila Pa 1976). 1996;21(24):2900-7. Epub 1996/12/15. doi: 10.1097/00007632-199612150-00023. PubMed PMID: 9112715.

15. Walton DM, Pretty J, MacDermid JC, Teasel RW. Risk Factors for Persistent Problems Following Whiplash Injury: Results of a Systematic Review and Meta-analysis. Journal of Orthopaedic & Sports Physical Therapy. 2009;39(5):334-50. doi: 10.2519/jospt.2009.2765. PubMed PMID: 19411766.

16. Kapoor S, Shaw WS, Pransky G, Patterson W. Initial patient and clinician expectations of return to work after acute onset of work-related low back pain. J Occup Environ Med. 2006;48(11):1173-80. Epub 2006/11/14. doi: 10.1097/01.jom.0000243401.22301.5e. PubMed PMID: 17099454.

17. Schultz IZ, Crook J, Meloche GR, Berkowitz J, Milner R, Zuberbier OA, et al. Psychosocial factors predictive of occupational low back disability: towards development of a return-to-work model. Pain. 2004;107(1-2):77-85. Epub 2004/01/13. doi: 10.1016/j.pain.2003.09.019. PubMed PMID: 14715392.

18. Schultz IZ, Crook J, Berkowitz J, Milner R, Meloche GR. Predicting return to work after low back injury using the Psychosocial Risk for Occupational Disability Instrument: a validation study. J Occup Rehabil. 2005;15(3):365-76. Epub 2005/08/27. doi: 10.1007/s10926-005-5943-9. PubMed PMID: 16119227.

19. Shaw WS, Pransky G, Patterson W, Winters T. Early disability risk factors for low back pain assessed at outpatient occupational health clinics. Spine (Phila Pa 1976). 2005;30(5):572-80. Epub 2005/03/02. doi: 10.1097/01.brs.0000154628.37515.ef. PubMed PMID: 15738793.

20. Iles RA, Davidson M, Taylor NF, O'Halloran P. Systematic review of the ability of recovery expectations to predict outcomes in non-chronic non-specific low back pain. Journal of Occupational Rehabilitation. 2009;19(1):25-40. PubMed PMID: 19127345.

21. Thomas E, Silman AJ, Croft PR, Papageorgiou AC, Jayson MI, Macfarlane GJ. Predicting who develops chronic low back pain in primary care: a prospective study. Bmj. 1999;318(7199):1662-7. Epub 1999/06/18. doi: 10.1136/bmj.318.7199.1662. PubMed PMID: 10373170; PubMed Central PMCID: PMCPMC28145.

22. Lang J, Ochsmann E, Kraus T, Lang JW. Psychosocial work stressors as antecedents of musculoskeletal problems: a systematic review and meta-analysis of stability-adjusted longitudinal studies. Soc Sci Med. 2012;75(7):1163-74. Epub 2012/06/12. doi: 10.1016/j.socscimed.2012.04.015. PubMed PMID: 22682663.

23. Poiraudeau S, Rannou F, Le Henanff A, Coudeyre E, Rozenberg S, Huas D, et al. Outcome of subacute low back pain: influence of patients' and rheumatologists' characteristics. Rheumatology (Oxford). 2006;45(6):718-23. Epub 2005/12/27. doi: 10.1093/rheumatology/kei231. PubMed PMID: 16377729.

24. Walton DM, Macdermid JC, Giorgianni AA, Mascarenhas JC, West SC, Zammit CA. Risk factors for persistent problems following acute whiplash injury: update of a systematic review and meta-analysis. Journal of Orthopaedic & Sports Physical Therapy. 2013;43(2):31-43. PubMed PMID: 23322093.

25. Palmlöf L, Skillgate E, Alfredsson L, Vingård E, Magnusson C, Lundberg M, et al. Does income matter for troublesome neck pain? A population-based study on risk and prognosis. J Epidemiol Community Health. 2012;66(11):1063-70. Epub 2012/03/14. doi: 10.1136/jech-2011-200783. PubMed PMID: 22412154.

26. Buscemi V, Chang W-J, Liston MB, McAuley JH, Schabrun SM. The role of perceived stress and life stressors in the development of chronic musculoskeletal pain disorders: A systematic review: The Journal of Pain. 2019, pp. No Pagination Specified.; 2019.

27. Dixon AN, Gatchel RJ. Gender and Parental Status as Predictors of Chronic Low Back Pain Disability: A Prospective Study. Journal of Occupational Rehabilitation. 1999;9(3):195-200. doi: 10.1023/A:1021353818130.

28. Singer J, Gilbert JR, Hutton T, Taylor DW. Predicting outcome in acute low-back pain. Can Fam Physician. 1987;33:655-9. Epub 1987/03/01. PubMed PMID: 21263854; PubMed Central PMCID: PMCPMC2218390.

29. Sterling M, Jull G, Vicenzino B, Kenardy J, Darnell R. Physical and psychological factors predict outcome following whiplash injury. Pain. 2005;114(1-2):141-8. Epub 2005/03/01. doi: 10.1016/j.pain.2004.12.005. PubMed PMID: 15733639.

30. Sterling M, Jull G, Kenardy J. Physical and psychological factors maintain long-term predictive capacity post-whiplash injury. Pain. 2006;122(1-2):102-8. Epub 2006/03/11. doi: 10.1016/j.pain.2006.01.014. PubMed PMID: 16527397.

31. Sterling M, Hendrikz J, Kenardy J. Similar factors predict disability and posttraumatic stress disorder trajectories after whiplash injury. Pain. 2011;152(6):1272-8. Epub 2011/03/15. doi: 10.1016/j.pain.2011.01.056. PubMed PMID: 21396780.

32. Goldsmith R, Wright C, Bell SF, Rushton A. Cold hyperalgesia as a prognostic factor in whiplash associated disorders: a systematic review. Manual Therapy. 2012;17(5):402-10. PubMed PMID: 22464187.

33. Agnello A, Brown T, Desroches S, Welling U, Walton D. Can we identify people at risk of non-recovery after acute occupational low back pain? Results of a review and higher-order analysis. Physiotherapy Canada. 2010;62(1):9-16. doi: 10.3138/physio.62.1.9. PubMed PMID: 105126216. Language: English. Entry Date: 20100409. Revision Date: 20150820. Publication Type: Journal Article.

34. Heneweer H, Aufdemkampe G, van Tulder MW, Kiers H, Stappaerts KH, Vanhees L. Psychosocial variables in patients with (sub)acute low back pain: an inception cohort in primary care physical therapy in The Netherlands. Spine (Phila Pa 1976). 2007;32(5):586-92. Epub 2007/03/06. doi: 10.1097/01.brs.0000256447.72623.56. PubMed PMID: 17334295.

35. Schiøttz-Christensen B, Nielsen GL, Hansen VK, Schødt T, Sørensen HT, Olesen F. Long-term prognosis of acute low back pain in patients seen in general practice: a 1-year prospective follow-up study. Fam Pract. 1999;16(3):223-32. Epub 1999/08/10. doi: 10.1093/fampra/16.3.223. PubMed PMID: 10439974.

36. van Oostrom SH, Monique Verschuren WM, de Vet HC, Picavet HS. Ten year course of low back pain in an adult population-based cohort--the Doetinchem cohort study. Eur J Pain. 2011;15(9):993-8. Epub 2011/03/25. doi: 10.1016/j.ejpain.2011.02.007. PubMed PMID: 21429779.

37. Hussain SM, Urquhart DM, Wang Y, Dunstan D, Shaw JE, Magliano DJ, et al. Associations between television viewing and physical activity and low back pain in community-based adults: A cohort study. Medicine (Baltimore). 2016;95(25):e3963. Epub 2016/06/24. doi: 10.1097/md.0000000000003963. PubMed PMID: 27336896; PubMed Central PMCID: PMCPMC4998334.

38. Kääriä S, Laaksonen M, Rahkonen O, Lahelma E, Leino-Arjas P. Risk factors of chronic neck pain: a prospective study among middle-aged employees. Eur J Pain. 2012;16(6):911-20. Epub 2012/02/18. doi: 10.1002/j.1532-2149.2011.00065.x. PubMed PMID: 22337254.

39. Kvalheim S, Sandven I, Hagen K, Zwart JA. Smoking as a risk factor for chronic musculoskeletal complaints is influenced by age. The HUNT study. Pain. 2013;154(7):1073-9. Epub 2013/04/30. doi: 10.1016/j.pain.2013.03.015. PubMed PMID: 23623251.

40. Dai Y, Huang J, Hu Q, Huang L, Wu J, Hu J. Association of Cigarette Smoking with Risk of Chronic Musculoskeletal Pain: A Meta-Analysis. Pain Physician. 2021;24(8):495-506. Epub 2021/11/19. PubMed PMID: 34793634.
