# Supplementary material for: The biopsychosocial factors associated with development of chronic musculoskeletal pain. An umbrella review and meta-analysis of observational systematic reviews
Source: PLoS One. 2024 Apr 1;19(4):e0294830. doi: 10.1371/journal.pone.0294830 (PMC10984407; doi:10.1371/journal.pone.0294830)
Supplement: S6 Table — (DOCX) [file pone.0294830.s008.docx]

**S9 Table.** Risk of bias assessment with AMSTAR-2 [1]

| Item No. | **Agnello [2]** | **Buscemi [3]** | **Chou [4]** | **Dai [5]** | **Fayad [6]** | **Goldsmith [7]** | **Iles [8]** | **Jadhakhan [9]** | **Lang [10]** | **Pincus [11]** | **Struyf [12]** | **Walton (2009) [13]** | **Walton (2013) [14]** |
| --- | --- | --- | --- | --- | --- | --- | --- | --- | --- | --- | --- | --- | --- |
| **1** | X | ✓ | ✓ | ✓ | ✓ | ✓ | ✓ | ✓ | ✓ | ✓ | ✓ | ✓ | ✓ |
| **2** | X | ✓ | X | ✓ | X | X | X | ✓ | X | X | ✓ | X | X |
| **3** | ✓ | X | X | ✓ | ✓ | ✓ | ✓ | ✓ | ✓ | ✓ | ✓ | ✓ | ✓ |
| **4** | X | X | X | ✓ | ✓ | ✓ | ✓ | ✓ | ✓ | ✓ | ✓ | ✓ | ✓ |
| **5** | X | ✓ | X | ✓ | ✓ | ✓ | ✓ | ✓ | ✓ | ✓ | X | X | X |
| **6** | ✓ | ✓ | X | ✓ | X | ✓ | X | ✓ | X | ✓ | X | X | X |
| **7** | X | ✓ | X | ✓ | X | ✓ | ✓ | ✓ | X | X | ✓ | ✓ | ✓ |
| **8** | ✓ | ✓ | ✓ | ✓ | ✓ | ✓ | ✓ | ✓ | ✓ | ✓ | ✓ | ✓ | ✓ |
| **9** | ✓ | ✓ | X | ✓ | X | ✓ | X | ✓ | ✓ | X | ✓ | ✓ | ✓ |
| **10** | ✓ | ✓ | X | X | X | ✓ | X | X | X | X | X | X | X |
| **11** | ✓ | NA | X | ✓ | NA | NA | NA | NA | ✓ | NA | NA | ✓ | ✓ |
| **12** | ✓ | NA | ✓ | ✓ | NA | NA | NA | NA | X | NA | NA | ✓ | ✓ |
| **13** | ✓ | ✓ | X | ✓ | X | ✓ | X | X | X | X | X | ✓ | ✓ |
| **14** | ✓ | X | X | ✓ | X | ✓ | ✓ | ✓ | X | ✓ | X | ✓ | ✓ |
| **15** | ✓ | NA | X | ✓ | NA | NA | NA | NA | ✓ | NA | NA | ✓ | ✓ |
| **16** | ✓ | ✓ | ✓ | ✓ | ✓ | ✓ | ✓ | ✓ | ✓ | ✓ | ✓ | X | ✓ |
| Confidence | **CL** | **L** | **CL** | **H** | **CL** | **L** | **CL** | **L** | **CL** | **CL** | **L** | **L** | **L** |

Confidence: critically low (CL), low (L), moderate (M), high (H)
Shaded rows are considered critical domains for adequately controlling risk of bias
Description of Items: 1. Research questions and inclusion criteria based on PICOS; 2. Protocol registered before commencement of the review; 3. Explained selection of studies included; 4. Provides evidence of adequate search strategy; 5. At least two reviewers involved in study selection; 6. At least two reviewers perform data extraction; 7. Justification provided for excluding individual studies; 8. Describes included studies in adequate detail; 9. Risk of bias adequately assessed for included studies; 10. Reported on sources of funding for included studies; 11. Appropriate meta-analysis methods used (if performed); 12. Assessed risk of bias impact on results of meta-analysis (if performed); 13. Accounts for risk of bias of individual studies when interpreting results; 14. Provides satisfactory explanation of heterogeneity of results (if present); 15. Assessed impact of publication bias on quantitative synthesis (if performed); 16. Reports and mitigates conflicts of interest for performing the review (if any)

1. Shea BJ, Reeves BC, Wells G, Thuku M, Hamel C, Moran J, et al. AMSTAR 2: a critical appraisal tool for systematic reviews that include randomised or non-randomised studies of healthcare interventions, or both. BMJ. 2017;358:j4008. doi: 10.1136/bmj.j4008.

2. Agnello A, Brown T, Desroches S, Welling U, Walton D. Can we identify people at risk of non-recovery after acute occupational low back pain? Results of a review and higher-order analysis. Physiotherapy Canada. 2010;62(1):9-16. doi: 10.3138/physio.62.1.9. PubMed PMID: 105126216. Language: English. Entry Date: 20100409. Revision Date: 20150820. Publication Type: Journal Article.

3. Buscemi V, Chang W-J, Liston MB, McAuley JH, Schabrun SM. The role of perceived stress and life stressors in the development of chronic musculoskeletal pain disorders: A systematic review: The Journal of Pain. 2019, pp. No Pagination Specified.; 2019.

4. Chou R, Shekelle P. Will this patient develop persistent disabling low back pain? Jama. 2010;303(13):1295-302. Epub 2010/04/08. doi: 10.1001/jama.2010.344. PubMed PMID: 20371789.

5. Dai Y, Huang J, Hu Q, Huang L, Wu J, Hu J. Association of Cigarette Smoking with Risk of Chronic Musculoskeletal Pain: A Meta-Analysis. Pain Physician. 2021;24(8):495-506. Epub 2021/11/19. PubMed PMID: 34793634.

6. Fayad F, Lefevre-Colau MM, Poiraudeau S, Fermanian J, Rannou F, Wlodyka Demaille S, et al. [Chronicity, recurrence, and return to work in low back pain: common prognostic factors]. Annales de Readaptation et de Medecine Physique. 2004;47(4):179-89. PubMed PMID: 15130717.

7. Goldsmith R, Wright C, Bell SF, Rushton A. Cold hyperalgesia as a prognostic factor in whiplash associated disorders: a systematic review. Manual Therapy. 2012;17(5):402-10. PubMed PMID: 22464187.

8. Iles RA, Davidson M, Taylor NF, O'Halloran P. Systematic review of the ability of recovery expectations to predict outcomes in non-chronic non-specific low back pain. Journal of Occupational Rehabilitation. 2009;19(1):25-40. PubMed PMID: 19127345.

9. Jadhakhan F, Evans DW, Falla D. The role of post-trauma stress symptoms in the development of chronic musculoskeletal pain and disability: A systematic review. Eur J Pain. 2023;27(2):183-200. Epub 2022/11/02. doi: 10.1002/ejp.2048. PubMed PMID: 36317593.

10. Lang J, Ochsmann E, Kraus T, Lang JW. Psychosocial work stressors as antecedents of musculoskeletal problems: a systematic review and meta-analysis of stability-adjusted longitudinal studies. Soc Sci Med. 2012;75(7):1163-74. Epub 2012/06/12. doi: 10.1016/j.socscimed.2012.04.015. PubMed PMID: 22682663.

11. Pincus T, Burton AK, Vogel S, Field AP. A Systematic Review of Psychological Factors as Predictors of Chronicity/Disability in Prospective Cohorts of Low Back Pain. Spine. 2002;27(5):E109-E20. PubMed PMID: 00007632-200203010-00017.

12. Struyf F, Geraets J, Noten S, Meeus M, Nijs J. A multivariable prediction model for the chronification of non-traumatic shoulder pain: A systematic review. Pain Physician. 2016;19(2):1-10. PubMed PMID: 608013657.

13. Walton DM, Pretty J, MacDermid JC, Teasel RW. Risk Factors for Persistent Problems Following Whiplash Injury: Results of a Systematic Review and Meta-analysis. Journal of Orthopaedic & Sports Physical Therapy. 2009;39(5):334-50. doi: 10.2519/jospt.2009.2765. PubMed PMID: 19411766.

14. Walton DM, Macdermid JC, Giorgianni AA, Mascarenhas JC, West SC, Zammit CA. Risk factors for persistent problems following acute whiplash injury: update of a systematic review and meta-analysis. Journal of Orthopaedic & Sports Physical Therapy. 2013;43(2):31-43. PubMed PMID: 23322093.
